# Identification, Gene Structure, and Expression of *BnMicEmUP*: A Gene Upregulated in Embryogenic *Brassica napus* Microspores

**DOI:** 10.3389/fpls.2020.576008

**Published:** 2021-01-11

**Authors:** Fariba Shahmir, K. Peter Pauls

**Affiliations:** Department of Plant Agriculture, University of Guelph, Guelph, ON, Canada

**Keywords:** microspore embryogenesis, Brassica napus, Arabidopsis, transcription factor, bZIP, gene expression, chloroplast localization

## Abstract

Microspores of *Brassica napus* can be diverted from normal pollen development into embryogenesis by treating them with a mild heat shock. As microspore embryogenesis closely resembles zygotic embryogenesis, it is used as model for studying the molecular mechanisms controlling embryo formation. A previous study comparing the transcriptomes of three-day-old sorted embryogenic and pollen-like (non-embryogenic) microspores identified a gene homologous to *AT1G74730* of unknown function that was upregulated 8-fold in the embryogenic cells. In the current study, the gene was isolated and sequenced from *B. napus* and named *BnMicEmUP* (*B. napus* microspore embryogenesis upregulated gene). Four forms of *BnMicEmUP* mRNA and three forms of genomic DNA were identified. *BnMicEmUP2,3* was upregulated more than 7-fold by day 3 in embryogenic microspore cultures compared to non-induced cultures. *BnMicEmUP1,4* was highly expressed in leaves. Transient expression studies of *BnMicEmUP3::GFP* fusion protein in *Nicotiana benthamiana* and in stable *Arabidopsis* transgenics showed that it accumulates in chloroplasts. The features of the BnMicEmUP protein, which include a chloroplast targeting region, a basic region, and a large region containing 11 complete leucine-rich repeats, suggest that it is similar to a bZIP PEND (plastid envelope DNA-binding protein) protein, a DNA binding protein found in the inner envelope membrane of developing chloroplasts. Here, we report that the *BnMicEmUP3* overexpression in *Arabidopsis* increases the sensitivity of seedlings to exogenous abscisic acid (ABA). The *BnMicEmUP* proteins appear to be transcription factors that are localized in plastids and are involved in plant responses to biotic and abiotic environmental stresses; as well as the results obtained from this study can be used to improve crop yield.

## Introduction

A striking characteristic of plants is that a wide variety of cells can be induced to develop into embryos. Microspore embryogenesis is one of the best-known examples of asexual embryo development in plants. In tissue culture, a mild stress can redirect microspores from their normal path of development, that leads to mature pollen formation, to the development of a haploid embryos, and ultimately to mature plants with a haploid number of chromosomes (Wędzony et al., 2009; Soriano et al., 2013; Humphreys and Knox, 2015). The haploid embryos or plants can be treated with colchicine to produce doubled haploid plants (Pauls, 1996; Seguí-Simarro and Nuez, 2008). Doubled haploid production from microspore (immature pollen) culture provides a faster method for developing homozygous plants than conventional selfing and selection and can improve the efficiency of plant breeding for a large number of plants, including dicot and monocot species (Pauls, 1996; Germanà, 2011; Humphreys and Knox, 2015; Shariatpanahi and Ahmadi, 2016).

Zygotic embryogenesis in plants is difficult to study because the process occurs within the ovule. In addition, it is difficult to study early stages of embryogenesis because the quantities of embryo tissue for cellular and molecular analyses are limited. *In vitro* haploid embryogenesis is considered an important tool for research of early embryo development, because the pattern of development during microspore embryogenesis is very similar to zygotic embryogenesis at many levels and because the liquid culture medium makes it easy to observe and manipulate the various stages of embryogenesis (Prem et al., 2012; Soriano et al., 2013). It is possible to produce thousands of embryos from microspore cultures, thus providing sufficient amounts of material for molecular and cellular studies of embryogenic processes in plants. Specifically, microspore embryogenesis in *Brassica napus* has been established as a model system for studies of early events in embryogenesis because of the simplicity and efficiency of this system (Schulze and Pauls, 1997; Seguí-Simarro and Nuez, 2008; Dubas et al., 2013; Soriano et al., 2013).

Several molecular and biochemical studies have demonstrated that microspore and zygotic embryos share key regulatory proteins and the expression of a number of transcription factors (Perry et al., 1999; Boutilier et al., 2002; Joosen et al., 2007; Soriano et al., 2013, 2014). *B. napus* microspore-derived embryo cultures were used to identify genes that are involved in the initiation of embryo development in plants. Comparative gene expression studies using embryogenic and non-embryogenic cells in *B. napus*, have resulted in the identification of several genes specifically expressed in embryogenic cells. Gene expression research in *Brassica* began with subtractive hybridization studies (Hattori et al., 1998; Tsuwamoto et al., 2007) and has continued with microarray analyses (Boutilier et al., 2002; Malik et al., 2007, 2008; Whittle et al., 2010). Hattori et al. (1998) performed a subtractive hybridization study using 4-day responsive and non-responsive microspore cultures to identify the upregulated genes in responsive cultures. This study identified and tested the function of two transcription factor genes, BNM2 and BABYBOOM (BBM). BNM2 was expressed during all stages of microspore and zygotic embryogenesis, especially during the storage accumulation period. However, BBM was expressed only during the early stages of microspore and zygotic embryogenesis in root and flower buds (non-seed tissues; Boutilier et al., 2002; Soriano et al., 2013). Genes expressed in microspore cultures have also been identified by a differential PCR technique. Custers et al. (2001) identified a gene family member of CLAVATA3/ESR (CLE) called CLE19 that is a member of a family expressed in meristem initials (Wang and Fiers, 2010; Xu et al., 2015) that also plays a role in the development of globular and heart stage microspore and zygotic embryos (Fiers et al., 2004).

Chan and Pauls (2006) found that the expression of the ROP GTPase BnRop9 was 2.5-fold higher in the embryogenic cells than in the pollen-like cells sorted from induced microspore cultures. In addition, they used *Arabidopsis* microarray cDNAs from the AR12K array [11,960 unique express sequence tags (EST) sequences including more than 6,000 genes] to identify genes upregulated in embryogenic microspore cultures after heat shock (Chan, 2006; Pauls et al., 2006). This study showed that 120 transcripts were upregulated, at least 2-fold, in embryogenic cells compared to non-embryogenic cells sorted from induced microspore cultures. These genes were classified into 13 groups with known functions and one group with unknown function. The genes of unknown function were upregulated more than 3–8-fold in embryogenic cells compared to pollen-like cells including a gene homologous to the *Arabidopsis* gene, *AT1G74730*, that has a motif of unknown function and was upregulated 8-fold in the embryogenic cells. The current work was initiated to confirm its upregulation in embryogenic microspore cells and gain an understanding of the function of the protein it encodes, in general.

In this work, we identify and characterize a gene that is differentially expressed during the switch from pollen development to microspore-derived embryo development, named *BnMicEmUP* (*B. napus* microspore embryogenesis upregulated gene). Our results show that *BnMicEmUP* encodes a unique protein, targeted to chloroplasts and containing a nucleotide-binding domain and a leucine zipper (bZIP)-like domain, similar to PEND (plastid envelope DNA-binding; Sato et al., 1998) proteins. It is preferentially expressed during microspore embryo induction and in expanded leaves and increases seedling sensitivity to exogenously applied abscisic acid (ABA).

## Materials and Methods

### Plant Material

*Brassica napus* L. cv. Topas plants were used throughout the experiments in this study. Seeds of *B. napus* were sown in soil in 24 cell-planting trays in a growth room at 20°C/18°C (day/night) under a 16 h photoperiod for microspore embryogenesis. Plants were fertilized with 1-1-1 ratio of nitrogen, phosphorous, and potassium and watered every 3 days. Once plants passed the three-leaf stage, they were transplanted to plastic pots (20 cm diameter, 25 cm height). Plants that were at the flower bud formation stage 45 days after sowing (DAS) of growth served as donors for flower bud collection and microspore isolation.

Unopened flower buds (2.0–3.0 mm in length) were selected from donor plants. The buds were surface sterilized in 75% ethanol for 30 s and then in 5.6% sodium hypochlorite bleach for 10 min. The sterilized buds were rinsed, three times, with sterile distilled water and homogenized in a blender with 30 ml cold 13% sucrose solution. The suspension was filtered consecutively through a 64 μm then 40 μm Nitex nylon sieves (Tetko Co., Elmsford, NY, United States). The filtrate was centrifuged for 5 min at 200 × *g*. The dark green supernatant was discarded and the pellet of microspores was re-suspended in wash solution (13% sucrose) and re-centrifuged. The procedure was repeated three times. The final pellet was re-suspended in full NLN medium (Nitsch and Nitsch, 1967; Lichter, 1982). The density of the microspores was adjusted to 8 × 10^4^ microspores/ml and 10 ml of the microspore suspension was dispensed into Petri plates (150 mm wide, 10 mm deep). The microspores were incubated at constant 30°C (inductive temperature) or 25°C (non-inductive temperature) in the darkness. The cultures were monitored from 0 to 5 days of culture (initiation phase microspore embryogenesis); each day, six plates were sampled for RNA extraction from responsive (30°C) and nonresponsive cultures (25°C).

### Isolation of the *BnMicEmUP* Gene and Southern Blotting

DNA was extracted from first leaves of 3-week old plants using CTAB method (Doyle, 1991). The genomic DNA was quantified using a Nanodrop ND1000 Spectrophotometer (Nanodrop Technologies, Inc., Wilmington, DE, United States). Approximately 50 μg of genomic DNA was digested with restriction enzymes *Xba*I and *Xho*I (New England Biolabs, Beverly, MA, United States). The digested DNA was separated on a 1.5% (w/v) agarose, 1 X TBE ethidium bromide gel at 40 V for 24 h and the results were photographed. The DNA was transferred to charged nylon membranes and left overnight (Roche Diagnostic, Laval, QC, Canada). Probes were prepared from pTopo2.1-*BnMicEmUP3* templates using a PCR DIG probe synthesis kit (Roche Diagnostics, Laval, QC, Canada). Hybridization was done using the DIG Easy Hyb Kit (Roche Diagnostics, Laval, QC, Canada). The hybridized probes were immuno-detected with anti-digoxigenin-AP, Fab fragments (Roche Diagnostics, Laval, QC, Canada) and then visualized by exposing Kodak X-OMAT film (Kodak Inc., Rochester, NY, United States) to the membrane treated with CDP-star (Roche Diagnostics, Laval, QC, Canada).

### RNA Extraction, Analysis, and Reverse Transcription to cDNA

Total RNA samples from 0 h, 1, 3, 5, and 7 days induced (30°C, embryogenic) and non-induced (25°C, non-induced) cultured microspores were isolated using glass bead disruption (Bio 101, Vista, CA, United States) and the modified RNeasy Midi kit (Qiagen), including on-column DNase digestion. RNA also was isolated from young leaves, old leaves, stem, buds, and root by using the RNeasy Midi kit. To remove genomic DNA from all RNA samples they were treated with Turbo DNAse, according to the manufacturer’s specifications (Ambion, Austin, TX, United States). The quality of the extracted RNA was determined with a Nanodrop-1000 (Thermo Fisher Scientific, Wilmington, DE, United States) by measuring the 260/280 ratio. The quality was tested by gel-electrophoresis through 1% agarose containing ethidium bromide (EtBr) at 80 V to visualize the 28S and 18S rRNA bands with a BIO-Rad Gel Doc System. The first strand of cDNA was synthesized from 1 μg of total RNA using the superscript first strand cDNA synthesis kit, according to manufacturer’s instructions (Invitrogen) or a SMART™ RACE cDNA Amplification Kits (Clontech, Palo Alto, CA, United States) were utilized, according to manufacturer’s instructions.

### Primer Design

To amplify *BnMicEmUP* genes from *B. napus*, the primer were designed based on the *AT1G74730* gene sequence available at the TAIR^1^ and *Brassica* ESTs (Genbank accession number: CN729138 and CN737378) with high sequence homology to *Arabidopsis thaliana*, which were found with Computational Biology and Functional Genomics Laboratory (CBFGL). The ESTs and *AT1G74730* cDNA sequences were aligned with CLUSTALW version 1.81^2^ and primers were designed for homologous regions with Primer3Plus (Untergasser et al., 2007) to amplify homologous cDNAs from *Brassica* cDNA (Supplementary Table 1). To isolate the upstream and downstream non-coding regions of *BnMicEmUP*, a SMART™ RACE cDNA Amplification Kit (Clontech, Palo Alto, CA, United States) was utilized, according to manufacturer’s instructions. In brief, total RNA was obtained from seedlings and induced microspore cultures on day 1, 2, and 3 and primers were designed from conserved regions of different forms of *BnMicEmUP* (Supplementary Table 1).

### Sequence Analysis and Conserved Domain Search

All sequencing results obtained were analyzed with BLAST at NCBI.^3^ To determine the number gene versions that exist in *Brassica*, the clones were aligned with CLUSTALW version 1.81 (Chenna et al., 2003).^4^ As a result of this alignment, a reliable set of SNPs between different forms of genes was discovered and a consensus sequence was generated.

An amino acid sequence comparison and similarities search was performed by BLAST in NCBI/N1H^5^ using the non-redundant database (nr) and in ExPASy.^6^ The search of conserved domains was carried out by NCBI BLAST and the protein sequences were compared with the Conserved Domain Database (CDD)^7^ as well. Although the CDD search included a search in Pfam (the database of protein domains), it does not integrate as many databases as InterProScan. Therefore, a search using InterProScan and the SMART database was performed to identify possible trans-membrane helices in the protein and signal peptides. To predict protein structure, the Phyre2 server was used (Kelley and Sternberg, 2009).

### Phylogenetic Analyses

Phylogenetic and molecular evolutionary analyses of the *BnMicEmUP* gene family were conducted using Workbench, version 3.0.2 (CLC bio, Aarhus, Denmark) software. Translated protein sequences were aligned and 60-amino acid regions containing putative homeodomains were chosen for phylogenetic analysis. They were imported into the *CLC* Workbench (CLC Bio, Aarhus, Denmark) program and globally aligned by CLUSTALW. A phylogenetic tree was generated with neighbor-joining methods, and bootstrap values were calculated.

### Gene Localization

Two fusion protein constructs, *BnMicEmUP3-GFP* and *AtBnMicEmUP-GFP*, were prepared to test the protein localization. To produce a fusion of *BnMicEmUP3* or *AtBnMicEmUP* with the green fluorescent protein (GFP; Siemering et al., 1996), fragments for the coding sequences of *BnMicEmUP3* or *AtBnMicEmUP* were amplified by PCR from clones containing the genes (*BnMicEmUP3* and *AT1G74730* with the forward primer (5'-GTCGTCGGCGCTCCAATATGGATCCAAGGAGATATAACAATGGCT-3') and specific reverse primers (5'-TGGATGGACTTCAAGAAGCTGACATGGGCAAGGGCGAGAAACTGTTC-3'). The reverse primers were designed to attach the 5'GFP sequence to 3' *BnMicEmUP3* gene with two design features. Firstly, it contained 15 nucleotides of 5' end of the GFP sequence and 15 nucleotides of the 3' end of the *BnMicEmUP3* sequence. Secondly, it was designed to remove the stop codon from the 3' end of the *BnMicEmUP3* gene. This primer was also used as the forward primer for GFP amplification, which was amplified from pCAMBIA 1302 (gene fusion reverse and GFP fusion forward: GCAGAGCTCATCTTCACTTGTAGAGTTCATCCA). The two amplified gene products were fused using the gene forward specific primer containing a *BamH*I site and ribosome binding site and GFP reverse primer containing *Sac*I. The *BnMicEmUP3-GFP* and *AtBnMicEmUP-GFP* fusion fragments were subcloned into *BamH*I-*Sac*I-digested pBI121 (Clontech) under control the CaMV35S promoter to generate pBIN35S::*BnMicEmUP3-GFP* and pBIN*35S*::*AtBnMicEmUP-GFP* plasmids. These were introduced into *Agrobacterium tumefaciens* strain EHA105.

### Chloroplast Isolation

Chloroplasts were isolated from transgenic *Arabidopsis* plants expressing *BnMicEmUP3*-GFP and *AtBnMicEmUP*-GFP and from wild type plants, following Takabe’s protocol (Takabe et al., 1979). The leaves (10 g) were homogenized in 0.5 M sucrose. The homogenate was filtered through eight layers of clean cheesecloth and centrifuged at 50 × *g* for 10 min. The pellets were resuspended in 4 ml of ice-cold 0.5 M sucrose. The pellet containing chloroplasts was examined with the confocal microscopy for GFP expression.

### Microscopic Study

To observe GFP fluorescence, isolated chloroplasts were observed immediately under an upright confocal laser scanning microscopy (CLSM; Leica DM RE microscope connected to a Leica TCS SP2, Leica, Germany). Images were processed using Leica Confocal Software (LCS, version 2.61).

### Gene Expression Level Assays Using Quantitative Real-Time PCR

**Figure 1:**
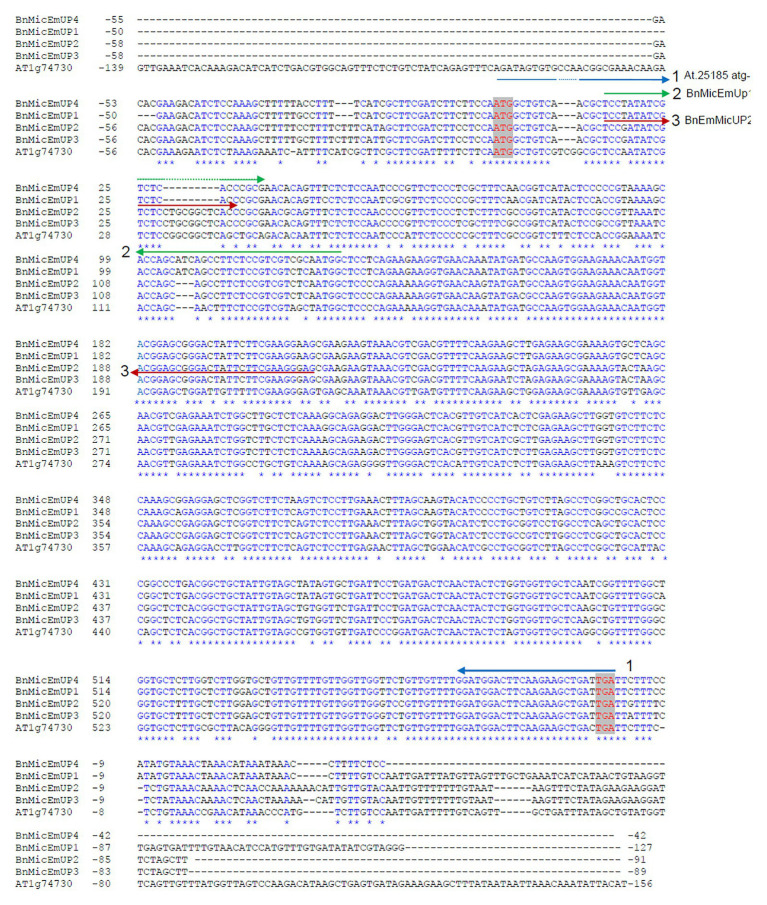
Comparison of nucleotide sequences of three forms of *Brassica napus BnMicEmUP* genes with the *Arabidopsis AT1G74730* gene. Yellow box (intron); un-highlighted region (the 5' un-translated and 3' un-translated regions); and white background (polymorphisms).

**Figure 2:**
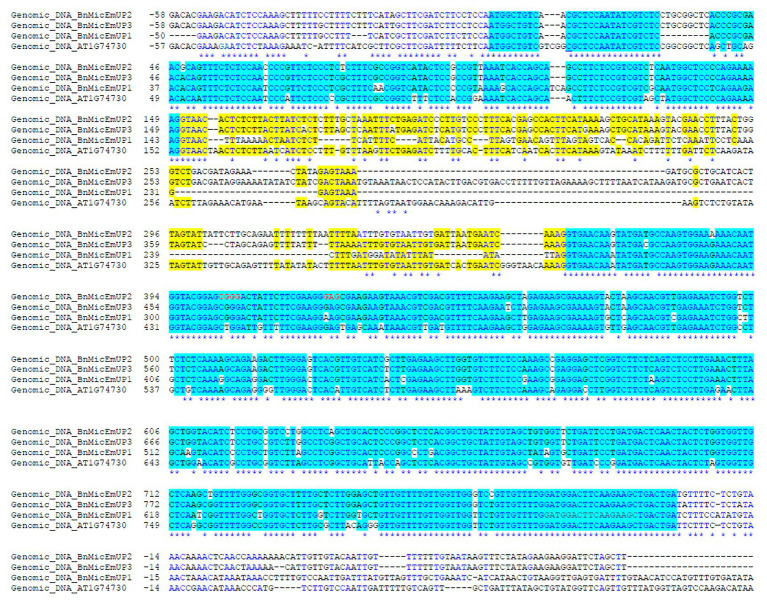
Comparison of the four forms of the *BnMicEmUP* cDNAs. The predicted 5'UTRs and 3'UTRs are shown in black and red sequences indicate the start and stop codons. Primers used for RT-PCR experiments are indicated with horizontal arrows. These primers were used to isolate and sequence two main classes of *BnMicEmUPs* namely: and *BnEmMicUP1,4* (2) and *BnEmMicUP2,3* (3; Supplementary Table 1).

To investigate the involvement of *BnMicEmUP2,3* gene expression in microspore embryogenesis of *B. napus*, comparative gene expression studies were done using isolated RNA from responsive (30°C) and nonresponsive (25°C) microspore cultures at different time points. Gene expression was analyzed by real-time PCR (RT-PCR) by using gene-specific primers designed for nucleotide deletion regions in *BnMicEmUP1,4* (without deletion) and *BnMicEmUP2,3* (with deletion; Figures 1, 2). Primers were designed to amplify either a *BnMicEmUP1* and/or a *BnMicEmUP4* fragment with a smaller size. The primers were: 5 prime (5-CCGATATCGTCTCCTGCGGCTC-3) and 3 prime (5-CGGGACTATTCTTCGAAGGGA-3) to produce a 160 bp or a larger size (200 bp) band, or 5 prime (5-CCTATATCGTCTCACCCGCGAA-3) and 3 prime (5-CATCAGCCTTCTCCGTCGTCGC-3) for *BnMicEmUP2* and/or *BnMicEmUP3* (Supplementary Figure 6). The optimal annealing temperature (TA) for each primer set was determined using the BioRad thermocycler. Real-time PCR was performed in 25 μl reactions with 1X IQ SYBR Green Supermix (Bio-Rad Laboratories) and 0.25 μM of each primer. An optimized volume of cDNA solution (100 ng) was used for the amplification of each gene. To reduce pipetting errors, master mixes were prepared for triplicate reactions (3 × 25 μl) for each standard and gene. Twenty microliter of the reaction mixture was pipetted into wells of a 96-well plate, followed by 5 μl of a 100 ng concentration cDNA solution into each well except for a blank which served as the “no template” control. The plates were sealed with microseal film, centrifuged, and placed on the optical reaction block of the BioRad Real-Time Detection System. Quantitative real-time PCR (qRT-PCR) protocols were set up using the iCycler iQ real time detection system software (BioRad). The same qPCR protocol was used for the two genes *BnMicEmUP2,3*. The following PCR program was used to amplify all genes: 5 min at 95°C followed by 40 cycles of 30 s at 95°C, 30 s at 65°C, and 30 s at 72°C, followed by 4°C hold.

To calculate alterations in transcription levels of target gene, standard curves with log input amount and CT values were applied for target gene and elongation factor 1-alpha (ef1alpha) for each sample using the iCycler iQ™ real time detection software (BioRad). For normalization, the amount of a target gene in each sample was divided by the amount of ef1alpha in the same sample. The normalized value was standardized by a control sample of each experiment (e.g., microspore in 0 day).

The analyses were performed on RNA isolated from three biological replications including three separate microspore cultures from 0 h, 1, 3, 5, and 7 days induced (30°C, embryogenic) and non-induced (25°C, non-induced) cultured microspores, and three preparations of vegetative tissue cDNA.

### Statistical Analyses

All statistical analyses were performed using GraphPad Prism 5.0 (GraphPad Software Inc., San Diego, CA, United States) and Excel. Data were analyzed with either paired Student’s *t*-test or one-way ANOVA to assess the changes in expression level of genes in different time during microspore culture and different tissue of *B. napus*. A probability level of *p* ≤ 0.05 was considered significant. All data are shown as mean ± standard error of mean (SEM). The confocal images were obtained as single optical sections and all images (*Arabidopsis* seedling leaf cells) shown in figures are representative of at least 20 cells from two independent lines.

### Preparation of Binary Vector Constructs With *AtBnMicEmUP2,3* Gene

The plasmids of pBI12135s::*AtBnMicEmUP* and pBI12135s::*BnMicEmUP3* (pBI121; Jefferson et al., 1987), containing coding region of *A. thaliana* and *B. napus* for *BnMicEmUP2,3*, were constructed to generate overexpressed lines. The *BnMicEmUP2,3* and *AtBnMicEmUP* genes were also cloned into the plant expression vector pFGC5941 (GenBank accession no AY310901),^8^ which forms a double stranded RNA (dsRNA) transcript under the control of the CaMV 35S promoter to generate constitutive gene silencing lines (RNAi lines). The RNAi construct was prepared using one highly gene-specific (unique) fragment, which was isolated from nucleotide positions 57–217. These plasmids were introduced into *A. tumefaciens* strain EHA105 (Hood et al., 1993). *Arabidopsis* plants were transformed with the constructs by the floral dip method (Clough and Bent, 1998). T0 seeds were selected on selection media as follows: Over-expressed lines produced with pBI121 were selected on kanamycin (50 mg/ml). The expression of *AtBnMicEmUP* genes in the *Arabidopsis* transformants and control lines were evaluated by RT-PCR using *AtBnMicEmUP* specific primers (At.25185 57-57 and At.25185 57-217; Supplementary Table 1). The orientation of the final construct was confirmed by DNA sequencing, using a pBI121 sequencing primer (35S promoter and N-terminal) and a gene specific primer. Homozygous T4 plants were used in all experiments.

SALK T-DNA insertion seeds (SALK_ 027022 and SALK_ 034644) were received from *Arabidopsis* Biological Resource Center (ABRC). Homozygous plants were obtained by PCR using primers designed by the SIGnALwebsite (Supplementary Table 1).^9^

For ABA germination assays, seeds were grown on 1/2 MS medium containing 1.5% sucrose and 0, 0.5, 1.0, and 2.0 μM ABA (Sigma, St. Louis, MO, United States) for 5 days. Three experimental repeats were carried out, each involving one independent AtBnMicEmUP mutant line (*atbnmicemup*-SALK_ 027022), two independent silenced lines (*AtBnMicEmUP-RNAi1.1, AtBnMicEmUP-RNAi1.2*), two overexpressing lines (*35S::AtBnMicEmUP 1.4, 35S::AtBnMicEmUP1.6*), and WT.

## Results

### Gene Structure

#### Isolation of *B. napus* Homologs of the *Arabidopsis* Gene *AT1G74730*

Gene sequences with homology to the gene *AT1G74730* (Figure 1) were amplified from *B. napus* (cv. Topas) genomic DNA by PCR and from RNA by RT-PCR from induced (at 30°C) microspore cultures, buds, and young leaves using PCR primers (At.25185 atg-gta, At.25185 118-735, and At.25185 57-217). These primers were designed based on multiple sequence alignments of ESTs and genomic sequences for the AT1G74730 gene and its homolog *AT5G08050* in Arabidopsis (Supplementary Table 1). Three fragments were amplified with primers At.25185 atg-gta forward and At.25185 atg-gta reverse from *B. napus* genomic DNA and named *BnMicEmUP* (*B. napus* Microspore Embryogenesis UPregulated) genes. The three genomic clones of *BnMicEmUP* had sizes of 709, 803, and 863 bp (Figure 1 and Supplementary Table 2). Genomic DNA sequences of BnMicEmUP1, BnMicEmUP2, and BnMicEmUP3 were submitted to GenBank and were given the accession numbers HQ647330, HQ647331, and HQ647332, respectively.

The *BnMicEmUP* genes have very similar structures to *AT1G74730* and consist of two exons and an intron (Figure 1). The nucleotide similarities among the *BnMicEmUP* genes and with *AT1G74730* were highest in the exons (85–87%) but more variable for the intron (43–69%). Particularly, *BnMicEmUP3*, which contains the longest intron (269 bp), differed from *BnMicEmUP1* intron (121 bp) by a 7 bp indel plus multiple single nucleotide polymorphisms (SNPs). The identity between the *BnMicEmUP3* and *BnMicEmUP2* (209 pb) introns is high (89%), but these two forms have low identities to the *BnMicEmUP1* intron sequence (13%) which is the shortest intron (121 bp). The *BnMicEmUP3* intron is also the most similar to the *AT1G74730* intron (Figure 1 and Supplementary Table 2).

Most of the differences in the exons were SNPs, but a 3-nucleotide indel in exon 1 distinguished the *B. napus* genes from the *Arabidopsis* gene, and a 9 nucleotide insertion in *BnMicEmUP2* and *BnMicEmUP3* separated those forms from *BnMicEmUP1* and *BnMicEmUP4* (Figure 2). The cDNA sequences of *BnMicEmUP1* (588 bp), *BnMicEmUP2* (594 bp), *BnMicEmUP3* (594 bp), and *BnMicEmUP4* (588 bp) were submitted to GenBank and were given the accession numbers, HQ660218, HQ660217, HQ660216, and HQ660215, respectively. The nucleotide identities with *AT1G74730cDNA* were high (85–89%). The differences were mostly related to SNPs spread throughout the coding region. Most of the SNPs occurred at the first coding exon. Sequence analysis of the *BnMicEmUP* cDNA clones indicates that there are at least two classes of cDNAs, which differ primarily by a nine-nucleotide deletion at position 30 of the coding region of the genes (Figure 2). The cDNA types containing the nucleotide deletion include the *BnMicEmUP1* and *BnMicEmUP4* genes. *BnMicEmUP3* and *BnMicEmUP2* belong to the cDNA type with no nucleotide deletion. The nucleotide sequence identity was 83–91% between the two classes of *BnMicEmUP* cDNAs (Supplementary Table 2). Similarly, these two cDNA classes also contain two different upstream untranslated regions (5' UTR) of *BnMicEmUP* with 92% identity. *BnMicEmUP1* and *BnMicEmUP4* belong to one class and BnMicEmUP2 and BnMicEmUP3 belong to a second class. However, cloning and sequencing of 5' and 3' RACE products allowed isolation of the short 5' UTR region, which is one-third as long as the 5' UTR region of *AT1G74730* (54–59 bp vs. to 150 bp). The 5' UTR region of *BnMicEmUP* has 61–72% identity with the homologous region of *AT1G74730* in Arabidopsis. More extensive heterogeneity was observed among the 3' UTRs of the different *BnMicEmUPs*. The sequence similarity between the 3' UTRs of the two classes of BnMicEmUPs was 47% and between these classes and AT1G74730 was 41–55%. The similarities between the coding regions of different classes of BnMicEmUP cDNAs and the gDNAs allowed the matched pairs to be identified. The results indicate that because four forms of cDNA were detected, but only three forms gDNA were identified, there is a possibility that another form of gDNA exists, which corresponds with cDNA of BnMicEmUP4 (Supplementary Figure 1).

#### Protein Domain Characterization

**Figure 3:**
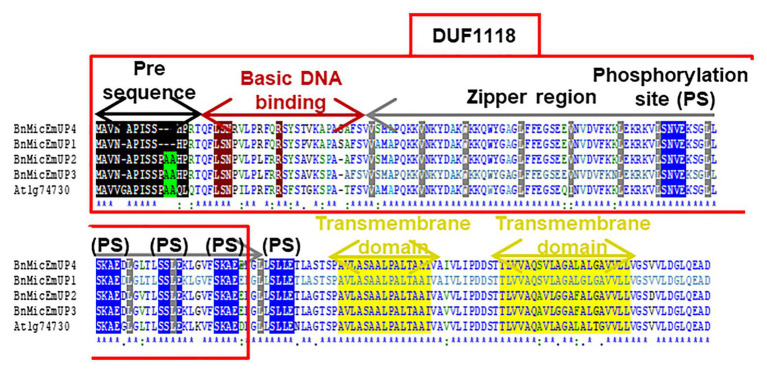
Comparison of four *B. napus* BnMicEmUP protein sequences with *Arabidopsis* AT1G74730. DUF1118 (domain unknown function) region is shown in the large box. Putative chloroplast transit peptides are highlighted in black and putative motifs (A/A) for chloroplast transit peptide cleavage sites are shaded in green. Basic DNA binding and leucine zipper (dimerization) domains are shaded in dark red and gray, respectively. Potential casein kinase II phosphorylation sites (S/TxxD/E) are shaded in blue. Putative transmembrane helix topologies of BnMicEmUPs are shaded in yellow.

**Figure 4:**
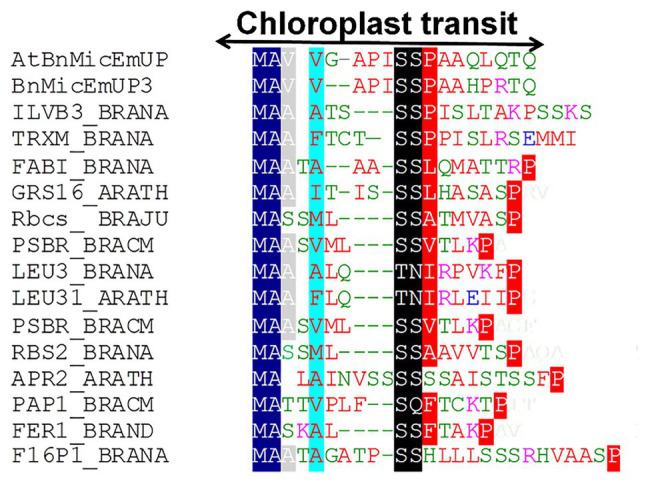
Alignment of predicted amino acid sequences of transit peptides of chloroplast targeted proteins with the AtBnMicEmUP and BnMicEmUP3 transit peptides. Species represented and the genes used to generate the sequence alignments are as follows: *B. napus* (BRANA); *B. rapa* subsp. campestris (BRACM); *A. thaliana* (ARATH); Ferritin-1 (Ferritin-1 Q96540.1); PSBR (Photosystem II 10 kDa polypeptide P49108.1); LEU3 (isopropylmalate dehydrogenase P29102.1); FABI (NADH-dependent enoyl-ACP reductase P80030.2); GSH1 (Glutamate – cysteine ligase CAD91713.1); ILVB1 (Acetolactate synthase 1 AAA62705.1); GRS16 (Monothiol glutaredoxin-S16 AEC09515.1); F16P1 (Fructose-1,6-bisphosphatase AAA82750.1); TRXM (Thioredoxin M-type Q9XGS0.1); Rubisco small subunit (Rbcs); Plastid lipid-associated protein 1 (PAP1); and 5'-adenylylsulfate reductase 2 (APR2). Conserved residues are highlighted in dark blue (block1), black (block2), and red (block 3). The consensus non-shared residues are colored according to: red, hydrophobic; purple, basic; green, hydrophilic; and blue, acidic.

The *BnMicEmUP3* ORF encodes a protein of 195 amino acids with an estimated molecular mass of 20.6 kDa. The amino acid composition of BnMicEmUP3 is 9.2% acidic, 21.5–22.5% polar, 10.7% basic, and 57.4–59.0% hydrophobic amino acids (Figures 3, 4) and the calculated *pI* = 9.3.^10^ A comparison of the four forms of putative BnMicEmUP proteins and AtBnMicEmUP (Figure 3) showed that they are 83–87% identical. A comparison of the BnMicEmUP amino acid sequence against a protein sequence database identified a region of the BnMicEmUP proteins sequence that is similar to the predicted Domain of Unknown Function 1118 (DUF1118; Figure 3).

Further searches for motifs in BnMicEmUP3 were performed using the domain alignments in PFAM (Bateman et al., 2002), SMART (Letunic et al., 2002), PRINTS (Attwood et al., 2002), and PROSITE databases. These analyses showed that BnMicEmUP contains a putative chloroplast transit peptide (cTP) in the N-terminus of the BnMicEmUP protein. Figure 4 shows that the putative BnMicEmUP cTP contains regions in its beginning, middle, and end sections that are well conserved in transit peptides sequences among diverse genes targeted to the chloroplast in *Brassica* and *Arabidopsis* species. In particular, all the sequences start with methionine and alanine (MA), followed by hydrophobic amino acids, like alanine or valine (A or V), a pair of serines (S), and terminate with proline P (Zybailov et al., 2008). The three-homology blocks are separated by different lengths of non-conserved sequences. A cleavage motif, consisting of two alanines (A/A), which is a unique cleavage site for cTPs (Zhang and Glaser, 2002) was also identified at the N-terminal sequences of the BnMicEmUP2 and BnMicEmUP3 putative protein sequences (Figure 3).

**Figure 5:**
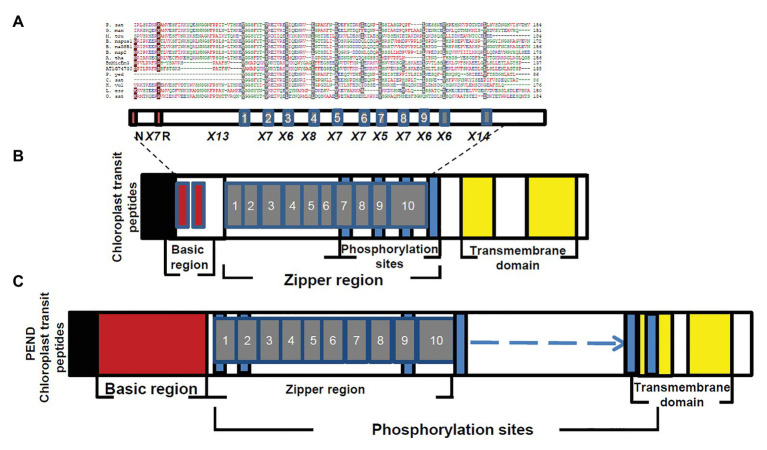
Comparison of BnMicEmUP3 protein to PEND proteins. **(A)** Alignment of PEND homologs to BnMicEmUP3 protein from various plant species. PEND sequences have been identified in various angiosperms by Terasawa and Sato, (2005). Sources of sequences: *P. sat* (*Pisum sativa*, PEND, genome: AB189736); *B. nap* (*B. napus*: GSBF1, cDNA: X91138); *B. nap1* (*B. napus*, genome: AB189734); *B. nap2* (*B. napus*, genome: AB189735); *A. tha* (*A. thaliana*, genome: AL094711); *G. max* (*G. max*, EST:BM308592); *M. tru* (*Medicago truncatula*, ESTs: BG644842); *L. esc* (*Lycopersicon esculentum*, ESTs: BE450861); *H. vul* (*Hordeum vulgare*, ESTs: AV833513); *O. sat* (*Oryza sativa*, genome: EST: AK106548); *C. sat* (*Cucumis sativus*, genome: AB189737); and *P. yed* (*Prunus yedoensis*, genome: AB189738) *BnMicEmUP3* (*B. napus*, genome: HQ660216). The consensus residues are colored according to: red, hydrophobic; purple, basic; green, hydrophilic; and blue, acidic. Highlighted residues show identical (dark red) or similar (gray) amino acids between BnMicEmUP bZIP domains and bZIP domains of PEND proteins. Additional common structural domains include a short pre-sequence (black), an N-terminal DNA-binding domain (dark red), central repeat domain (gray), and C-terminal transmembrane domains (yellow). **(B)** Phylogenetic tree of PEND homologs. The phylogenetic tree was constructed by the neighbor-joining method with 100 bootstrap replicates using CLC software. The numbers on branches show bootstrap confidence levels based on 100 bootstrap trials.

**Figure 6:**
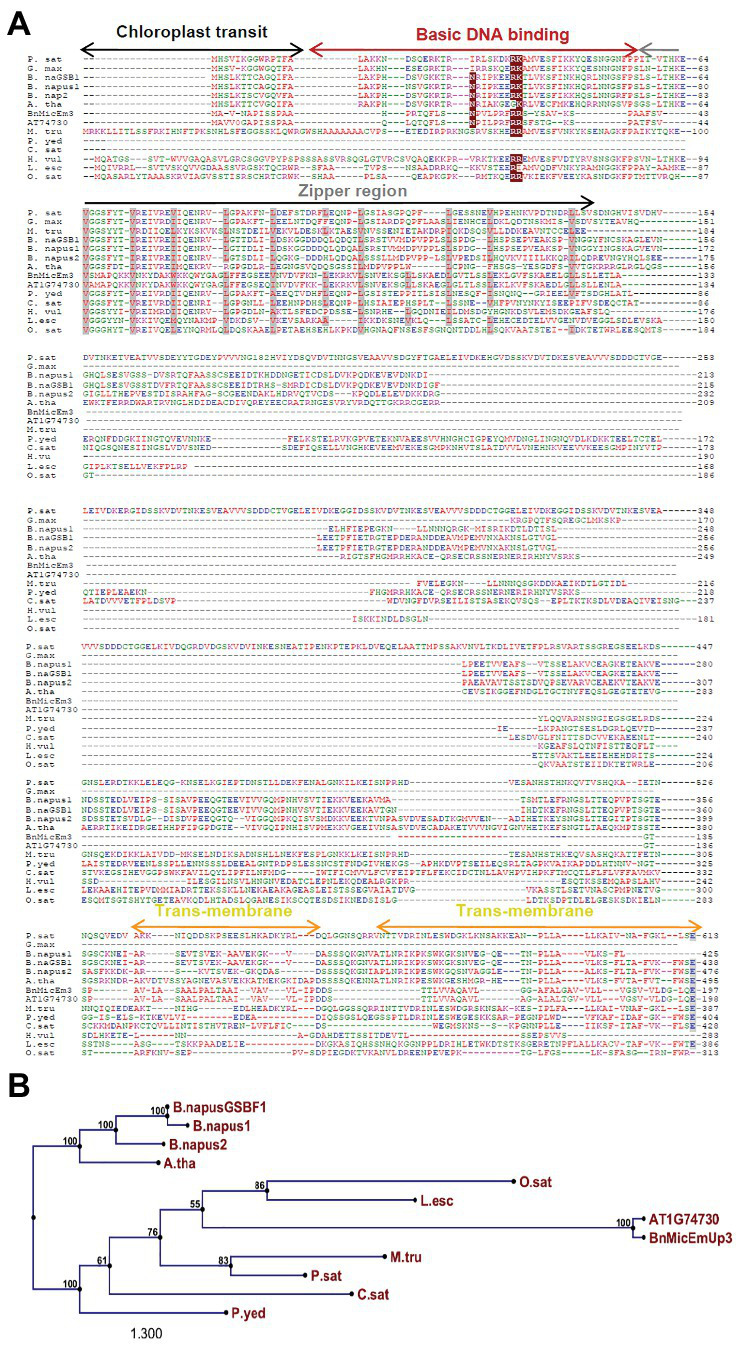
Structural features of BnMicEmUP3 compared to PEND proteins. **(A)** Alignment of the BnMicEmUP bZIP domain with selected bZIP domains of PEND proteins. Sources of sequences: *P. sat* (*Pisum sativa*, PEND, genome: AB189736); *B. nap* (*B. napus*: GSBF1, cDNA: X91138); *B. nap1* (*B. napus*, genome: AB189734); *B. nap2* (*B. napus*, genome: AB189735); *A. tha* (*A. thaliana*, genome: AL094711); *G. max* (*Glycine max*, EST: BM308592); *M. tru* (*Medicago truncatula*, EST: BG644842); *L. esc* (*Lycopersicon esculentum*, EST: BE450861); *H. vu l* (*Hordeum vulgare*, EST: AV833513); *O. sat* (*Oryza sativa*, genome: EST: AK106548); *C. sat* (*Cucumis sativus*, genome: AB189737); and *P. yed* (*Prunus yedoensis*, genome: AB189738) *BnMicEmUP3* (*B. napus*, genome: HQ660216). The consensus residues are colored according to red, hydrophobic; purple, basic; green, hydrophilic; and blue, acidic. Highlighted residues are identical (dark red) or similar (gray) amino acid residue between BnMicEmUP bZIP domains and bZIP domains of PEND protein. Similar structural domains include a short pre-sequence (black), a N-terminal DNA-binding domain (dark red), and a central repeat domain (gray). **(B)** Schematic diagram of the BnMicEmUP3 protein showing the locations of the chloroplast transit peptide (black), the basic DNA binding, zipper region (dark red), predicted transmembrane domains (yellow), and casein kinase II phosphorylation sites. **(C)** Schematic diagram of the PEND protein showing similar domains to BnMicEmUP3 including a chloroplast transit peptide, a basic DNA binding region, a zipper region, predicted transmembrane domains, and casein kinase II phosphorylation sites.

BnMicEmUP protein also contains DNA-binding domains that are most similar to those found in the basic leucine zipper (bZIP) family (PROSITE accession no ps00036). A common motif for bZIP proteins is a basic region at the N-terminus (between amino acids 17 and 69 or 125) that includes an invariant DNA-binding motif N-x_7_-R, followed by a repeat of four to ten Leu, which may be replaced by other hydrophobic residues such as isoleucine, alanine, and valine. An alignment of the BnMicEmUP DNA binding amino acid sequence with bZIP sequences from the plant transcription database^11^ shows the high conservation of the characteristic features of this group of transcription factors (Figures 5A, 6A) . Especially, the occurrence of N, at amino acid position 19 of the BnMicEmUP protein, is highly conserved in DNA binding bZIP family proteins (Figure 3). However, BnMicEmUP has a much longer stretch of intervening amino acids, between the putative DNA binding site and the start of the zipper region, that is found in typical bZIPs (Jakoby et al., 2002; Figure 3). Moreover, the leucine zipper of BnMicEmUP protein consists (initially) an octad, rather than a conventional heptad. The consensus repeat for BnMicEmUP is V/L X4-X8 V/L.

The essential features of the BnMicEmUP protein include a chloroplast-targeting region, a basic region, and a large repeat region containing 11 complete leucine-rich repeats. These features have been observed in members of the bZIP family called PEND (for plastid envelope DNA binding) proteins. This family of DNA binding proteins is found in the inner envelope membrane of developing chloroplasts and response to environmental changes (Terasawa and Sato, 2009). A multi alignment of PEND proteins from different species of angiosperms including: *P. sativa*, *B. napus*, *A. thaliana*, *G. max*, *M. truncatula*, *L. esculentum*, *H. vulgare*, *O. sativa*, *C. sativus*, *P. yedoensis*, with BnMicEmUP and AT1G74730 (Terasawa and Sato, 2005; Figures 5A,B) reveals that the basic region, zipper region, and transmembrane domains are conserved, but the central region is highly variable in these proteins. The occurrence of the essential features of PEND proteins in BnMicEmUP, including a possible chloroplast transit peptide, a DNA binding site, a leucine rich repeat region, and transmembrane domains (Figures 6A–C) supports the proposal that it can be considered to be a member of this unique protein family.

A protein analysis of the amino acid sequence for BnMicEmUP showed that this protein also contains several casein kinase II phosphorylation sites that correspond to the consensus S/TXXD/E, in the 89–129aa region (Figure 3). These four repeat sites include acidic residues aspartic acid (D) or glutamic acid (E), close to the targets of the phosphorylation, serine or threonine (S or T; Meggio and Pinna, 2003). The same analysis of the PEND protein from the Prosite database^12^ (Gasteiger et al., 2005; Hulo et al., 2006) showed that it also contains several casein kinase II phosphorylation sites (Figure 6C).

The protein secondary structure prediction by CLC (CLC bio, Aarhus, Denmark) showed that the BnMicEmUP protein has a characteristic *α*-helical structure in the region that contains the leucine-rich repeats (Supplementary Figure 2A), which is the main structural motif found in bZIP proteins (Jakoby et al., 2002). In addition, two putative trans-membrane domains were predicted by phyre2 (Kelley and Sternberg, 2009) that correspond to α-helix topologies for the same regions modeled by CLC (Figure 6). The overall structure predictions for the proteins coded by the four *Brassica* genes and the *Arabidopsis* gene were very similar. They all have extended C-terminal regions with some helical structure and end with two regions of strongly folded (alpha helix or beta strand) amino acids that are associated with transmembrane regions (Supplementary Figures 2B, 3).

**Figure 7:**
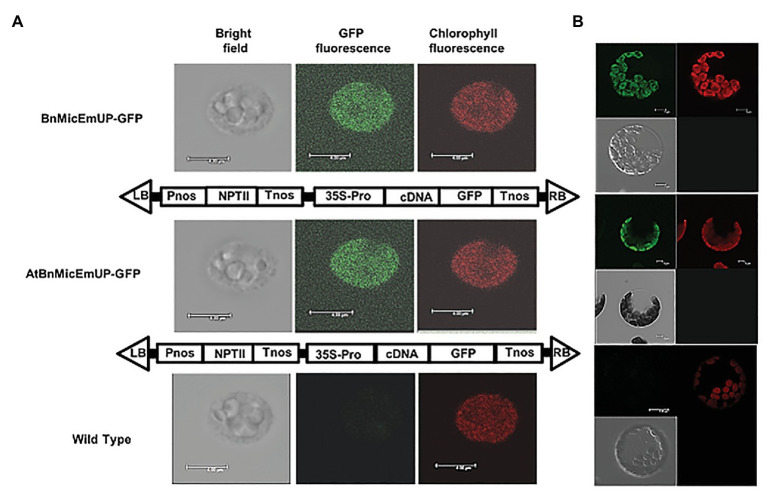
Targeting of *AtBnMicEmUP:GFP* and *BnMicEmUP3::GFP* to the chloroplast. **(A)** Chloroplasts were isolated from transgenic *Arabidopsis* transformed with *pBI12: AtBnMicEmUP::GFP* (top, diagram of the construct vector) and *pBI121::BnMicEmUP3::GFP* (middle, diagram of the construct vector). Right: chlorophyll fluorescence; middle: GFP fluorescence; and left: chloroplast in bright light. Scale bar = 4 μm. **(B)** Isolated protoplast. Right: chlorophyll fluorescence; left: GFP fluorescence; and left bottom: protoplast in bright light. Scale bar = 5 μm.

#### Analysis Elements in *AtBnMicEmUP* and *BnMicEmUP3* Promoter

To characterize specific elements within the AtBnMicEmUP3 promoter, a 580 bp sequence upstream of the coding region (nucleotide 28,078,401–28,079,001) was obtained from TAIR SeqViewer.^13^ A 539 bp sequence upstream of the co ding region for BnMicEmUP3 (22,077,826–22,078,365) was obtained from COGE^14^ (Chalhoub et al., 2014) analyzed with PlantCARE^15^ (Lescot et al., 2002) and PLACE database (Higo et al., 1999).^16^ The analysis for the *AtBnMicEmUP* promoter identified nine potential TATA sequences at 463, 437, 429, 411, 384, 353, 230, 180, and 155 bases upstream from the start of transcription. Four CAAT sequences were found at 35, 106, 264, and 320 bases upstream from the start codon (ATG). In the *BnMicEmUP3* promoter region, the analysis identified nine potential TATA sequences at 57, 135, 216, 349, 350, and 422 bases upstream from the start of transcription. Four CAAT sequences were found at 48, 76, 240, 317, 395 (−) bases upstream from the start codon (ATG). In addition to these core promoter elements, the analysis of the upstream sequence of *AtBnMicEmUP* and *BnMicEmUP3* identified a number of *cis*-regulatory elements that are associated with gene regulation by light, metabolism, fungal elicitors, and growth regulators. The functions of these predicted *cis*-acting elements are categorized in Supplementary Table 3 and their arrangement in the upstream region of *BnMicEmUP3* and *AtBnMicEmUP* is shown in Supplementary Figures 4, 5, respectively. The cis-acting regulatory elements involved in light responsiveness (G-box, GT1-motif, GA-motif, GAG-motif, AE-box, and BOX4) were found, and abscisic acid responsiveness (ABRE), a MeJA-responsiveness (CGTCA-motif and TGACG-motif), and auxin-responsiveness (TGA-box) elements all shared nucleotides centered at positions 461 and 410 in *BnMicEmUP3* and *AtBnMicEmUP*, respectively. The *BnMicEmUP3* and *AtBnMicEmUP* promoters also contain elements that are induced by physiological and environmental factors, including wounding (W box), fungal elicitors (ERE), heat shock (HSE), pathogen or stress response (TC-rich repeats), auxin (ARE), and light (G-box, GT1-motif). In addition, the promoter regions contain a MBS-MYB binding site, involved in drought-inducibility and a TGACG site, involved in the methyl jasmonate (MeJA)-responsiveness.

### Gene Localization

#### Bioinformatics-Based Prediction of Protein Sub-Cellular Localization

Eleven different programs were used to predict the sub-cellular localization of BnMicEmUP and AtBnMicEmUP proteins. The majority of the predictions placed these proteins, in either mitochondria or plastids, especially chloroplasts (eight programs; Supplementary Table 4). Seven of the 11 programs analyzed the N-terminal sequence rather than the whole protein. Six of these programs predicted chloroplast localization for BnMicEmUP with high reliability (87–96%). PSORT predicted thylakoid membrane localization for BnMicEmUP and a mitochondrial membrane localization as a second option. Two of the programs predicted mitochondrial localization for BnMicEmUP proteins. In particular, TargetP, a neural Web-based predictor (Emanuelsson et al., 2000), predicted chloroplast and mitochondrial localization with likelihood values of 0.65 and 0.52, respectively (Guda, 2006).

#### Targeting of BnMicEmUP Homologs to Chloroplasts

The subcellular localization of the BnMicEmUP protein was experimentally tested by creating constructs with *AtBnMicEmUP* or *BnMicEmUP3* tagged with GFP at their C-terminal. The majority of the chloroplasts isolated from transgenic *Arabidopsis* plants expressing *BnMicEmUP3::GFP* and *AtBnMicEm P*::*GFP* had a uniform green fluorescence (Figures 7A,B), suggesting that the *BnMicEmUP3*::*GFP* was dispersed throughout mature chloroplasts.

#### *BnMicEmUP* Copy Number

**Figure 8:**
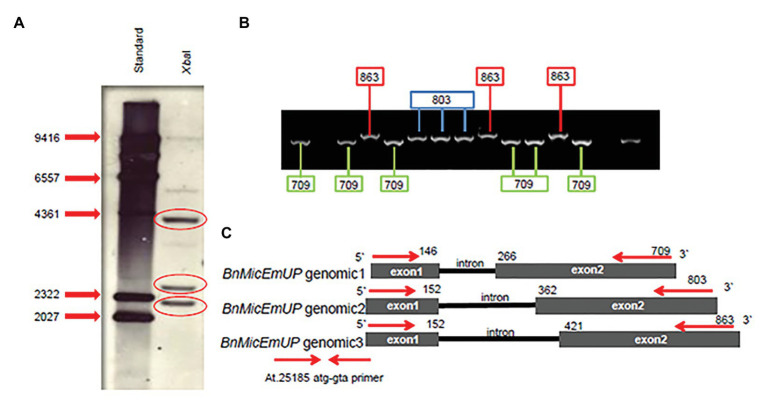
*BnMicEmUP* gene copy numbers. **(A)** Southern blot of *B. napus* DNA cut with restriction enzyme *Xba*I, probed with *BnMicEmUP3*. **(B)** PCR products derived from amplifications of individual colonies of PCR fragments into TOPO vectors using the At.25185 atg-gta primer. Cloning PCR fragments into TOPO vectors randomly picked from a plate and used as PCR templates contained fragments at three different discrete sizes of *BnMicEmUP* genomic DNA size (black line). The genomic organization of the *Brassica* gene is the same as the *Arabidopsis*, and the genomic DNA fragments are indicated with different colors. **(C)** The comparison of the *BnMicEmUP* gene structure with *Arabidopsis AT1G74730*. Three forms of the gene with different sizes are shown, comprised of two exons (black boxes) and one intron.

Southern blots of genomic DNA from young *B. napus* leaves digested with *Xba*I and probed with Dig-labeled *BnMicEmUP3* had three bands ranging in size from 2 kb to 4.3 Kb (Figure 8A). In addition, the genes were detected in genomic DNA by PCR with specific primers (At.25185 atg-gta) that resulted approximately 800 kb in size. When the PCR product was cloned into a TOPO cloning vector, three different clones, with similar, but distinguishable inserts were obtained that were 709, 803, and 863 bp in size (Figure 8B). These results indicate that there are at least three copies of the *BnMicEmUP* gene in the *B. napus* genome (Figure 8C).

**Figure 10:**
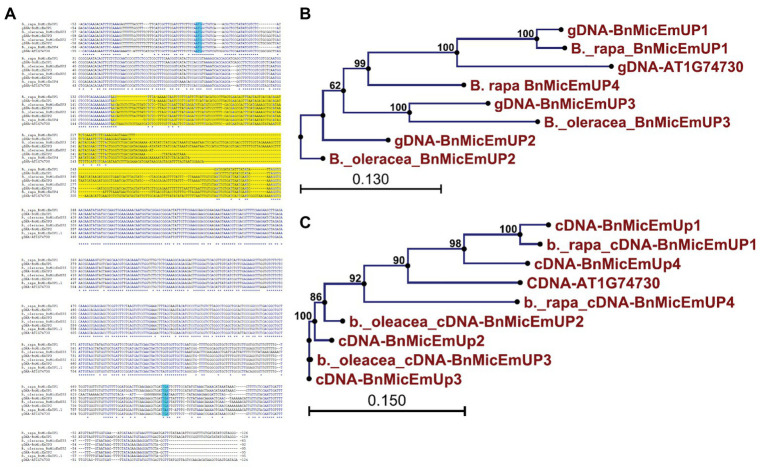
Comparative analysis of gene expression patterns of different forms of *BnMicEmUP* in induced and non-induced microspore cultures. **(A,B)** Expression of *BnMicEmUP2,3* and *BnMicEmUP1,4* from 24 to 110 h in induced (30°C, embryogenic) and non-induced (23°C, nonembryogenic) microspore cultures, values shown here relative to EF1alpha, and transcription levels of genes at day 0. **(C)** RNA quality evaluated by visualizing the RNA on a gel, showing two bright discrete bands that represent the 28S and 18S ribosomal species. **(D)** *B. napus* EF1alpha (housekeeping gene) as a positive control for cDNA quality. Error bars show variance (SE) among three biological replicates. Means with the same letter are not significantly different.

#### Genome Origins of the Three *BnMicEmUP* Homologs

**Figure 9:**
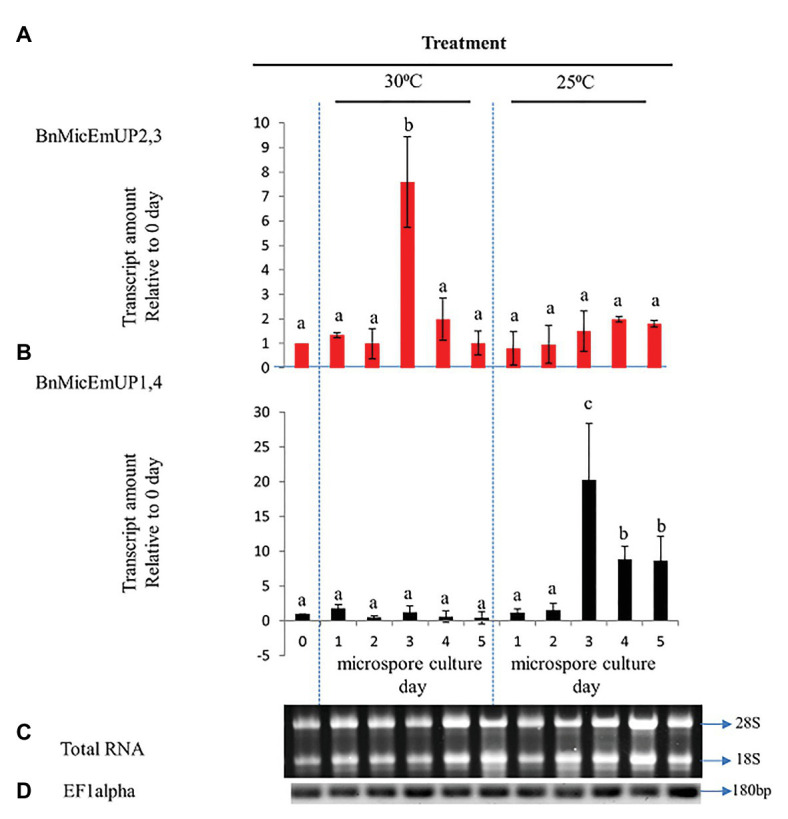
Sequence analysis of the *BnMicEmUP* genes from *B. napus* and its diploid progenitors, *Brassica rapa*, and *Brassica oleracea*. **(A)** Genomic DNA sequence alignment of the *BnMicEmUP* genes. **(B)** Phylogenetic tree of the genomic sequences of the *BnMicEmUP* genes. **(C)** Phylogenetic tree of the cDNA sequences of the *BnMicEmUP* genes. Sequences labeled with the blue color indicate start and stop codon and yellow color shows intron region.

*Brassica napus* is an allotetraploid (AACC) that originated by hybridization of two diploid progenitors, *Brassica rapa* (AA) and *Brassica oleracea* (CC; Nagahara, 1935). To study the genome origins of the *BnMicEmUP* homologs, they were BLASTed against the *B. rapa* and *B. oleracea* genomes. The *BnMicEmUP1* gene exhibited highest sequence similarity (*E*-value < 1) with a *BnMicEmUP1* homolog from *B. rapa*. Therefore, the *B. rapa* gene was named *BnMicEmUP1* (Figures 9A–C). Additionally, *BnMicEmUP2* and *BnMicEmUP3* were predicted to be paralogous to *B. olerace BnMicEmUP* genes (*E*-value < 1). The *BnMicEmUP2* and *BnMicEmUP3* genes cloned from *B. napus* with nine nucleotide insertions in intron did not have counterparts within the *B. rapa* genome. Another copy of a *BnMicEmUP* was identified in *B. rapa* (which is predicted to be homologous to the unidentified genomic DNA *BnMicEmUP4* (Figures 2, 9C).

**Figure 11:**
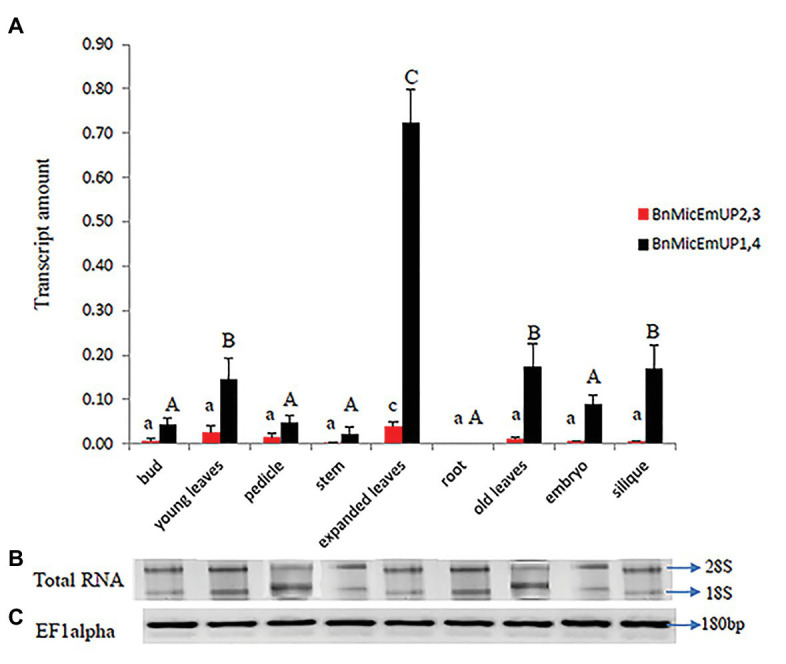
Real time PCR studies of *BnMicEmUP2,3* and *BnMicEmUP1,4* expressions in different tissues. **(A)** Comparison of the expression patterns of *BnMicEmUP2,3* and *BnMicEmUP1,4* in different tissues of *B. napus*. The transcription rates were expressed relative to the EF1alpha transcription rate. **(B)** RNA quality evaluated by visualizing the RNA on a gel, showed two bright discrete bands that represent the 28S and 18S ribosomal species. **(C)** *B. napus* Ef1alpha (housekeeping gene) expression was used as a positive control for cDNA quality. Error bars show variance (SE) among three biological replicates. Means with the same letter are not significantly different.

### Gene Expression Profile of *BnMicEmUP* Gene in *B.napus*

#### Microspore Culture

To investigate the specificity of *BnMicEmUP* gene expression during microspore culture, cDNA from RT-PCR was examined by RT-PCR in reactions containing *BnMicEmUP* primers and RNA from freshly isolated microspores from non-responsive (25°C) or responsive (30°C) microspores cultured for 5 days. RNA isolated at different times after microspore culture initiation over 5 days in culture at 30 °C (inductive) and 25°C (non-inductive) was of good quality according to a PCR assay of the cDNA produced from it and primers for EF1 alpha. A single band of the expected size (180 bp) was observed in all of the samples. In addition, separations of the total RNA from the samples all had clearly visible 28S and 18S rRNA species, indicating that the RNA was intact (Figures 10C,D). Supplementary Figure 5 shows that the primers for these forms are highly specific. The *BnMicEmUP1,4* primer amplified a band of 160 bp from the cloned *BnMicEmUp1and 4*-Topo2.1 but not the cloned *BnMicEmUp2* and *3*-Topo2.1. The *BnMicEmUp2,3* primers give a larger product (200 bp) only with *BnMicEmUp2,3* Topo2.1 clones (Supplementary Figure 6).

*BnMicEmUP2,3* and *BnMicEmUP1,4* genes showed different patterns of expression in induced microspores cultured at 30°C and non-induced microspores cultured at 25°C (Figures 10A,B). *BnMicEmUP2,3* expression levels were more than 7.6-fold higher in 3 day induced cultures compared to day 0 cultures, but the other three time points did not show such clear differences in *BnMicEmUP2,3* expressions (Figure 10A). One-way ANOVA showed that the difference between *BnMicEmUP2,3* expressions at day 3 was significantly (*p* < 0.05) higher than the levels of expression at 0, 1, 2, 4, and 5 days in responsive cultures. Although *BnMicEmUP2,3* expression levels were slightly higher than day 0 at day 1 and day 4, the differences were not statistically significant. No significant differences were detected between different time points for *BnMicEmUp1,4* expressions in responsive (30 °C) microspore cultures. However, three patterns of expression of *BnMicEmUP1,4* were observed in non-induced (25 °C) cultures. *BnMicEmup1,4* expression levels in non-induced cultures were statistically significantly higher after 3, 4, or 5 days compared to day 0 as well as between day 3 and all other time points. There was no significant difference in *BnMicEmUP1,4* expressions in microspore cultures between day 4 and day 5 at 25°C.

#### Organ-Specific Expression Profile of *BnMicEmUP*

*BnMicEmUP1,4* was highly expressed in every organ type and developmental stage except for roots, where it was not detectable (Figure 11A). In contrast, *BnMicEmUP2,3* showed very low expression levels, compared to *BnMicEmUP1,4* in all of the tested tissues (Figure 11A). The expression of *BnMicEmUP1,4* was 6-fold higher in expanded leaves compared to immature leaves. The ANOVA showed that the high level of *BnMicEmUP1,4* expressions in expanded leaves was significantly different from its expression in other parts the plant. Expression differences for *BnMicEmUP1,4* among buds, young and old leaves, embryo, and silique tissues were not significantly different and approximately one-seventh of the level seen in the expanded leaves but approximately 2-fold higher (significantly different) than the expression levels observed in the bud, pedicle, root, and embryo. *BnMicEmUP1, 4* expression differences among the bud, pedicle, root, and embryo tissues were not significantly different from each other (Figure 11A). RNA integrity (18s and 26s) and RNA quality (EF1 alpha, 180 bp) are described in Figures 11B,C.

#### BnMicEmUP Function

**Figure 12:**
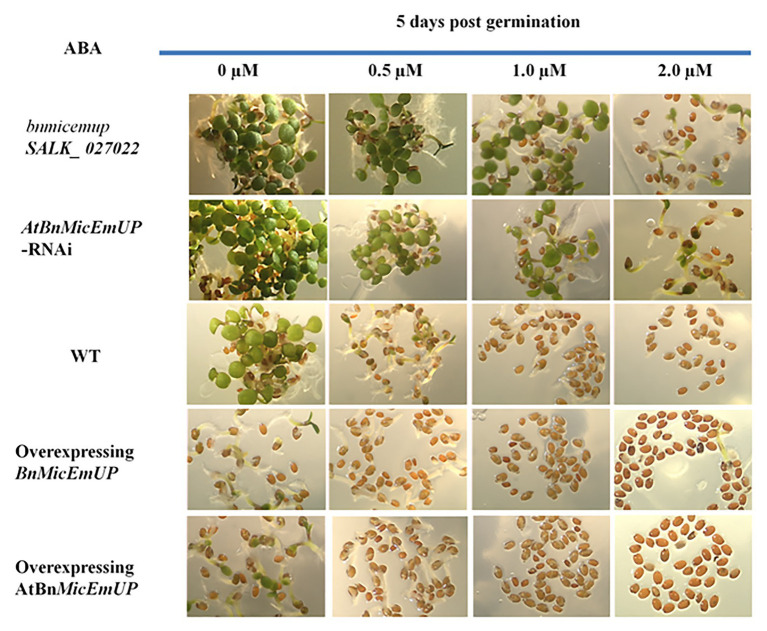
Effect of *AtBnMicEmUP3* expressions on development of *Arabidopsis* seedlings in presence of different levels of abscisic acid (ABA), 5 days post-germination. Seeds from one independent AtBnMicEmUP mutant lines (*atbnmicemup*-SALK_ 027022), two independent silenced lines (*AtBnMicEmUP-RNAi*), two independent overexpressing lines (*35S::AtBnMicEmUP3*), and WT were germinated on plates containing 0, 0.5, 1.0, and 2.0 μM ABA for 5 days. The figures show representative responses of the experiment that was conducted three times.

Transgenic *Arabidopsis* over expression lines with *BnMicEmUP3* under control of the cauliflower mosaic virus 35S promoter had delayed seedling development, compared to the wild type. Conversely, seedling development was accelerated in the knockdown (mutant and RNAi) *Arabidopsis* lines compared to wild type (Figure 12).

Germination of wild type and overexpressing lines on various levels of ABA (between 0 and 2 μM) delayed their seedling development. In contrast, seedling sensitivity to ABA was reduced in the knockdown lines. For example, both the wild type seedlings on media without ABA (control) and *BnMicEmUP3* knockdown lines on.5.0 μM ABA had similar numbers of green cotyledons, 5 days after transferring to germination medium, whereas, none of the *BnMicEmUP3* overexpressing lines and wild type lines were green on 5.0 μM ABA.

## Discussion

### Gene Copies

The present finding that *B. napus* contains multiple copies of *BnMicEmUP* homologs was expected because *B. napus* is an allotetraploid (AACC) that arose from hybridization of two diploid progenitors, *B. rapa* (AA) and *B. oleracea* (CC; Nagahara, 1935). In fact, information from recent *B. napus* sequencing efforts,^17^ made it possible to identify the genomic origins of the homologs of *BnMicEmUP* genes identified in the present work. The *BnMicEmUP2,3* homologs, with the nine nucleotide insertions, are most similar to *B. oleracea* sequences and, therefore, likely originated from the C genome, while the *BnMicEmUP1* homolog is most similar to the sequence from *B. rapa*. In addition, when the recently sequenced and annotated *B. napus* genome^18^ (Chalhoub et al., 2014) was searched for *BnMicEmUP* sequences four *BnMicEmUP* genes were found (Figure 9A). Therefore, the presence of the *BnMicEmUP4* genomic DNA was confirmed and the total number of *BnMicEmUP* genes, and their phylogenetic relationships are in agreement with the evolutionary history of *Brassica* species.

The greatest variability among the different copies of the *BnMicEmUP* gene was in the lengths of their introns. In contrast, the *BnMicEmUP*s exons are highly conserved. This is consistent with general findings in other organisms (Alberts et al., 2002). Although intron variations are not generally correlated with functional or developmental differences, they may affect levels of gene expression (Coulombe-Huntington and Majewski, 2007). Castillo-Davis et al. (2002) have shown that genes with shorter introns have higher levels of expression than genes with longer introns. Therefore, it is possible that variations in the intron lengths and sequences of *BnMicEmUP* genes are related to differential gene expression levels and expression patterns.

### Gene Expression

The current finding that the *BnMicEmUP2,3* homologs are highly upregulated during the first few days in embryogenic microspore cultures, confirms our previous finding that a transcript with homology to *AT1G74730* was upregulated in embrogenic microspore cells (Chan, 2006; Pauls et al., 2006) suggesting that these genes play a role in the differentiation and/or commitment to embryo development-specific. In contrast, the upregulation of BnMicEmUP1,4 homologs in non-responsive microspore cultures and other tissues, such as leaves, indicates that they are markers for vegetative tissues. Differential regulation was also reported for five members of plant ROP genes (Rho-GTPases) in various organs and during androgenesis in *B. napus*, including upregulation of *BnROP9* in embryogenic cells sorted from induced microspore cultures (Chan and Pauls, 2006).

Supporting evidence for the expression patterns for *BnMicEmUP* genes was obtained with the eFP browser, “a visualization tool for exploring publicly available microarray data sets” (Winter et al., 2007). The pictorial representations of gene expression that this tool (Supplementary Figure 7) confirmed that *AtBnMicEmUP* is highly expressed in expanded leaves, young leaves, and the globular embryo stage in *Arabidopsis* but, is weakly expressed in the roots and mature leaves, and is not expressed in ripened siliques. Perhaps, because the gene encodes a putative chloroplast-targeted protein, its high expression in expanded leaves is related to the relatively high numbers of chloroplasts that would be expected in growing and expanding leaf cells (Possingham and Saurer, 1969; Pyke, 1999).

No information about the expression of the *AtBnMicEmUP* during embryo initiation (day 0–5) is available in the eFP browser; however, high expression levels of the gene are shown for globular stage embryos, which occur 7 days after fertilization (Supplementary Figure 7B). These analyses also revealed that the level of expression of this gene was higher at day 7 than at day 15, when the embryos were at the heart and torpedo stages, respectively (Borkird et al., 1988). The high expression of *AtBnMicEmUP* in the globular stage of embryo development also supports the relationship between *AtBnMicEmUP* expression and chloroplast development because chloroplast biogenesis occurs during early globular stage embryogenesis (Tejos et al., 2010) and it has been suggested that the ability of cells to develop chloroplasts can be used as a marker for cell differentiation during embryogenesis. In addition, loss of chloroplast function in *Arabidopsis* leads to embryo lethality (Tzafrir et al., 2004).

Moreover, the eFP browser revealed that the expression of AtBnMicEmUP (homolog of *BnMicEmUP*) was downregulated by abiotic stresses, such as osmotic, heat, and UV stress. Interestingly, it was shown that heat stress (3 h at 38°C followed by recovery at 25°C) in seedlings and shoots downregulated AtBnMicEmUP after 3, 4, and 6 h of treatment (Winter et al., 2007). This contrasts with the current results that indicate that the BnMicEmUP2,3 genes in *Brassica* is highly upregulated by day 3 in embryogenic microspores after exposure to a mild heat stress. Perhaps, differences in heat treatments (i.e., 30°C in the current study vs. 38°C for the previous work) and tissues tested (i.e., microspores vs. seedlings) account for the different responses to heat stress. Together, the results suggest that expression of BnMicEmUP is regulated by stress.

### Promoter Analysis

The central regions of the *BnMicEmUP3* promoters have combinations of different light-responsive elements, stress-response elements, and hormone-response elements that allow for different levels of expression during plant growth and development and environmental conditions (Yamaguchi-Shinozaki and Shinozaki, 2005). They may also provide interesting links between specific developmental and stress responses. The multiple TATA (nine) and CAAT (four) box sequences found in *BnMicEmUP3*, which are defined as core promoter elements (Kozak, 1981), may function to promote high levels of transcription (Kiran et al., 2006). Multiple TATA boxes may also promote differential transcription, depending on the environmental conditions (Doyle and Han, 2001). In particular, the multiple TATA boxes in *BnMicEmUP3* promoters may be related to the stress inducibility of these genes (Bae et al., 2015). The ABRE elements found in the *BnMicEmUP3* gene promoters are important cis-acting elements in ABA-responsive genes that mediate gene expression responses to abiotic stress (Xu et al., 1996). The cis-acting elements that are responsive to jasmonic acid (Wasternack, 2007; Balbi and Devoto, 2008) in the *BnMicEmUP3* promoters may support the response of these genes to insect pests. The W box type elements that occur in *BnMicEmUP3* promoters are binding sites for WRKY transcription factors, which also have roles in gene expression responses to wounding associated with pathogenesis and herbivory (Hara et al., 2000). WRKY transcription factors are also known to modulate gene expression during a variety of developmental and physiological processes including embryogenesis (Lagacé and Matton, 2004), and stem cell maintenance in apical meristems (Rose et al., 2006).

The large number of light responsive cis-elements, such as the GT1 motif and G-Box, in the upstream sequences of *BnMicEmUP3* genes may indicate that they are regulated by light. However, these elements may also be an indication of sensitivity to stress since they also occur in the promoters of genes that respond to various environmental stressors and ABA (Williams et al., 1992; Komarnytsky and Borisjuk, 2003). Some light responsive cis-elements are found in promoters of genes that are not light-regulated (Kuno and Furuya, 2000). Therefore, the presence of LREs in the promoter of *BnMicEmUP3* is not enough to consider this gene as a photo-regulated gene. Nevertheless, it could be a link between the chloroplast localization of the protein and some roles of light in the stress induced microspore embryogenesis, since previous experiments with *B. napus* microspore cultures showed that embryo production was significantly promoted by growing the donor plants at high light (Thurling and Chay, 1984).

A very interesting finding was the extensive overlap of cis-acting regulatory elements for abscisic acid (ABRE), MeJA (CGTCA-motif and TGACG-motif), and auxin responsiveness (TGA-box) in one region of the promoter at position 461, next to circadian *cis*-elements. This region may have an important role in coordinating the expression of *BnMicEmUP3* in response to various signaling pathways, through competition for common promoter sites. Other work has shown that ABI3, an ABA responsive transcription factor, binds to both ABA and auxin response element (Nag et al., 2005).

### Characteristics of the BnMicEmUP Predicted Protein

The results presented here demonstrate that BnMicEmUP is a PEND protein with a short pre-sequence, an N-terminal DNA-binding domain (cbZIP), a central repeat domain, and a C-terminal transmembrane domain. The analysis of the polypeptide encoded by BnMicEmUP strongly indicated that it contains a putative bZIP domain, which makes it a member of one of the largest transcription factor (TF) families in plant genomes (Jakoby et al., 2002).

This assignment as a putative bZIP transcription factor was based on the identification of a basic DNA binding motif and leucine zipper motifs (Figures 3, 5A). Additionally, the bioinformatics and experimental evidence for the chloroplast localization of BnMicEmUP characterizes it as a cbZIP. A previous comparison of cbZIP domains with typical bZIP domains (Terasawa and Sato, 2005) showed that there were clear differences that related to variation in the structure of the central region that separates the DNA binding and leucine zipper motifs. For example, A PEND protein from pea, a cbZIP DNA-binding protein in the inner envelope of the chloroplast (Terasawa and Sato, 2005), contains a central region exceeding 20 amino acid residues (Sato et al., 1998). This variation in the central region was also reported in genes homologous to PEND, such as the GS-box binding factor 1 (GSBF1) of *B. napus* and GSBF1 homolog of *A. thaliana* (Sato et al., 1998). Similarly, the BnMicEmUP central region contains 13 amino acids, which is longer than the nine amino acid residues typically found in bZIP domains. Surprisingly, the common motif of basic DNA binding domains, consisting of invariant Asn and Arg residues (Miller, 2009), is not conserved in the PEND from pea (Sato et al., 1993) and its homologs (Sato et al., 1998), but it is conserved in the basic region of the BnMicEmUP protein. In accordance with a previous comparison of the amino acid sequences of bZIP proteins, which showed more similarities in their basic domains than in their leucine zippers (Vettore et al., 1998), the current study showed that leucine residues are replaced by other hydrophobic residues in many of the heptads in BnMicEmUP (Figure 5). In addition, a variation in the number of amino acids between the basic region and heptad repeat of hydrophobic amino acids has been observed in many members of the pea PEND (cbZIP) protein.

The transmembrane domains in the C-terminal end of the BnMicEmUP polypeptide were identified based on the occurrence of regions that contain ~20 hydrophobic residues. Such an arrangement of amino acids is necessary for the formation of a helix to span a biological membrane (Rost et al., 1995). A model for the BnMicEmUP peptide structure that is similar to PEND (Terasawa and Sato, 2009) has the C-terminal transmembrane domain of the processed protein inserted into the inner chloroplast membrane and the N-terminal DNA-binding domain oriented toward the stroma so that it can bind to chloroplast DNA.

The N-terminal end of the BnMicEmUP polypeptide also contains a signal peptide region as a distinct and separate region from the basic region (BR) that serves as either a nuclear localization signal (NLS; van der Krol and Chua, 1991) or chloroplast localization signal (Terasawa and Sato, 2005) in other proteins. The transit peptide in the N-terminus of AtBnMicEmUP identified by PSORT is only 15 amino acids long, which is shorter than most known chloroplast presequences (Heins et al., 1998; Keegstra and Cline, 1999). However, similar lengths of chloroplast protein presequences were reported for the PEND and E37 proteins (Terasawa and Sato, 2009). The three amino acid homology in the transit peptides of the BnMicEmUPs (Figure 5) has also been identified in the transit peptides of two nuclear-encoded chloroplast proteins, including chlorophyll a/b protein and the small subunit of ribulose-1,5-bisphosphate carboxylase (Rubisco; Karlin-Neumann and Tobin, 1986). Chloroplast import pre-sequences are cleaved during import and a putative processing site consisting of the A/A motif (Zhang and Glaser, 2002) was identified at the N-terminal end of the chloroplast presequence of the BnMicEmUP protein.

In agreement with the present results (C-terminal GFP/BnMicEmUP fusion protein), a study to identify proteins targeted to the plastid (Koo and Ohlrogge, 2002) indicated that AT1G74730 (AtBnMicEmUP) was localized in the chloroplast envelope, but a study by Friso et al. (2009) suggests that it is located in the thylakoid membrane. The absence of detectable levels of GFP fluorescence in other organelles indicated that the GFP/BnMicEmUP fusion protein was very efficiently targeted to chloroplasts. Yokoyama et al. (2016) recently suggested that two homologs of this same gene encode proteins (they called RIQ1 and RIQ2) that affect the organization of the light-harvesting complex II and grana stacking in *Arabidopsis*, however, did not comment on their DNA binding motifs.

The BnMicEmUP protein is not the only transcription factor located in the plastid. Other transcription factors, either PEND or non-PEND proteins, have been shown to be located in plastids. Examples of these proteins (Nakano et al., 2003): a plastid envelope DNA binding protein (PEND) from *P. sativum* containing a basic domain followed by leucine zipper motif (Thurling and Chay, 1984), proteins named PD1 and PD3 also from *P. sativum* (Sato et al., 1995). A protein designated as Plastid Transcription Factor 1 (PTF1) containing a basic helix-loop-helix (bHLH) motif from *A. thaliana* has also been suggested as a plastid transcription factor (Baba et al., 2001). The NtWIN4 protein, a bHLH motif containing a wound-induced protein from *N. tabacum*, has also been reported to be a transcription repressor with roles in adaption to biotic and abiotic stresses (Kodama and Sano, 2006). Kodama and Sano (2007) suggested that these proteins have evolved from eukaryotic nuclear transcription factors into plastid functional proteins. Furthermore, a current hypothesis is that these genes were altered evolutionarily to allow plants to cope with environmental stresses (Sato, 2001).

### Growth Arrest Mediated by BnMicEmUP

The delay in development observed in the present study for the constitutively expressing *35S*::*BnMicEmUP3* transgenic *Arabidopsis* seedlings supports the hypothesis that *BnMicEmUP3* expression inhibits plant growth and development. Interestingly, a similar delay in seedling growth was reported for *Arabidopsis* seedlings overexpressing ABSCISIC ACID INSENSITIVE 5 (*ABI5*), during the first 3 days after seed germination (Lopez-Molina et al., 2001; Garcia et al., 2008; Wang et al., 2013). Many studies have shown that the addition of endogenous ABA maintains seed dormancy, resulting in the inhibition of seed germination (Nambara and Marion-Poll, 2003; Leymarie et al., 2008; Rodríguez-Gacio et al., 2009). The similarity of the delay in seedling growth, therefore, suggests that ABA may be associated with *BnMicEmUP3* function and the finding that *BnMicEmUP* overexpression results in an enhanced response to ABA supported this conclusion.

BnMicEmUP is also structurally similar to ABI5, since both have elements of basic leucine zipper (bZIP) transcription factors. Moreover, both BnMicEmUP and ABI5 contain casein kinase phosphorylation sites, which may be phosphorylated by SnRK2s (Finkelstein et al., 2005; Nakashima et al., 2009). Furthermore, the identification of conserved ABA-responsive elements (ABRE; PyACGTGGC), which control ABA-regulated gene expression, in the promoter of *BnMicEmUP3* suggests that *BnMicEmUP3* is stress inducible and may itself be regulated by ABA.

### ABA May Be Associated With *BnMicEmUP* Function During the Induction Phase of Microspore Embryogenesis

Several lines of evidence suggest that an ABA signal may promote the observed upregulation of *BnMicEmUP2,3* during the induction phase of microspore embryogenesis. In particular, different kinds of stress, which would increase endogenous ABA levels (Sah et al., 2016), are widely applied as pretreatments in anther and microspore cultures of tobacco (Imamura and Harada, 1980), barley (van Bergen et al., 1999). It has also been shown that androgenesis is improved by the addition of exogenous ABA in wheat and tobacco (Kyo and Harada, 1986; Hu et al., 1995). Heat-stress induction of embryogenesis in microspore cultures of *B. napus* is associated with calcium release and alkalization of the microspore (Pauls et al., 2006). Consequently, an ABA signaling pathway may be directly or indirectly involved in the activation of the gene expression during microspore embryogenesis (Hays et al., 1999; Tsuwamoto et al., 2007). Furthermore, inhibition of ABA synthesis reduces regeneration efficiency in androgenesis (Hoekstra et al., 1997). In addition, the ABI3 gene, which regulates ABA responsiveness in plants (Brady et al., 2003), is a necessary embryogenesis factor (McCourt, 1999) and is considered a molecular marker gene for microspore embryogenesis in *B. napus* (Malik et al., 2007). Interestingly, a subtracted cDNA library composed of upregulated genes during androgenic initiation shows that these genes contain ABA-inducible *cis*-elements, such as the ABRE motif in their promoter regions (Tsuwamoto et al., 2007).

### Model for BnMicEmUP Protein Involvement in ABA-Mediated Transcriptional Regulation

**Figure 13:**
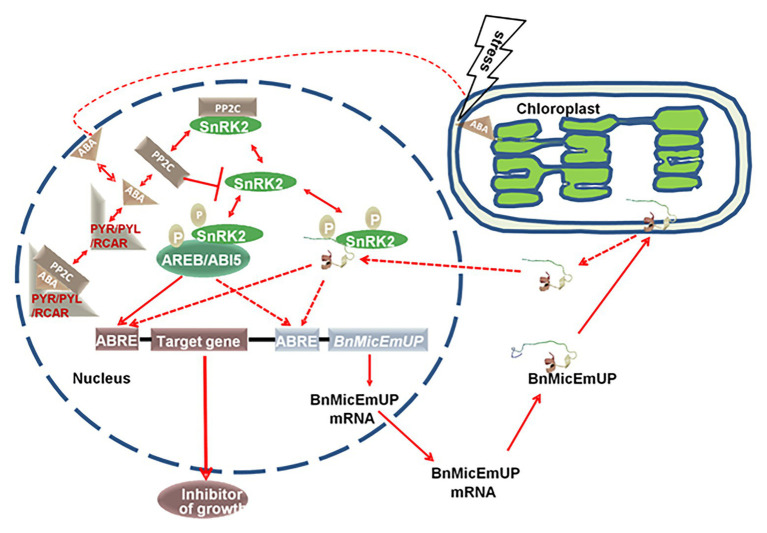
A proposed model for a dual targeting of BnMicEmUP protein and its involvement in ABA-mediated transcriptional regulation. BnMicEmUP protein is first sequestered to chloroplasts by transit through the outer membrane and is possibly associating with the inner membrane through interaction with the transmembrane domains. Stress leads to the release of BnMicEmUP and shuttling to the nucleus. In the nucleus, in the absence of ABA, PP2C inhibits autophosphorylation of a SnRK kinase by dephosphorylation. In the presence of ABA, PYR/PYL/RCAR binds ABA and sequesters PP2C. As a result, SnRK2 becomes auto-activated and can subsequently phosphorylate downstream factors, such as the ABREB/ABI5 or transcription factor or BnMicEmUP. AB15 or BnMicEmUP either could stimulate transcription of a downstream inhibitory protein or may stimulate its own transcription in a self-regulatory manner.

A model for BnMicEmUP involvement in ABA signaling is proposed in Figure 13. In this model, the signaling system includes: an ABA receptor (PYR/PYL/RCAR; Ma et al., 2009; Park et al., 2009), a type 2C protein phosphatase (Umezawa et al., 2009; Vlad et al., 2009; PP2C), a SNF1-related protein kinase (2SnRK2; Fujii and Zhu, 2009; Umezawa et al., 2010) and leucine zipper (bZIP) transcription factors as target of SnRK2. This system can regulate ABA-mediated ABRE-dependent gene expression either negatively or positively. The PP2C inactivates SnRK2 by *dephosphorylation* in absence of ABA, whereas, in the presence of ABA, PYR/PYL/RCAR binds to ABA, resulting in PP2C inhibition and SnRK2 activation. Then SnRK2 phosphorylates a basic-domain leucine zipper (bZIP) transcription factor (Cutler et al., 2010; Hubbard et al., 2010; Klingler et al., 2010; Raghavendra et al., 2010). In the model, BnMicEmUP functions as a transcription factor that is stored in chloroplasts. Stress causes it to be released from the plastid envelope and translocated to the nucleus where it functions as an ABRE-binding proteins (AREBs) that is regulated through phosphorylation mediated by SnRK2s (Finkelstein et al., 2005; Nakashima et al., 2009). Interestingly, *BnMicEmUP3* can function as an ABA-regulated gene because it has ABRE elements in its promoter that are targets for AREBs, such as *ABI5*. In this model *ABI5* and *BnMicEmUP3* have similar functions. Synergistic interactions between *ABI5* and several other bZIP ABA response factors have been reported (Reeves et al., 2011).

In summary, it can be proposed that the BnMicEmUP protein has two structural forms that act at different times and different locations. The complete structure of protein contains a putative cTP in the N-terminus and trans-membrane domains in C-terminus, analogous to a PEND protein, could be involved in sequestering and storage of the protein before activation in chloroplast. The second structure would be derived from the first after activation by a stress stimulus that is perceived by chloroplasts. It is proposed that the process involves movement of the sequestered protein from chloroplast to the nucleus, dimerization, and interaction with DNA, through the basic DNA binding motif, to act as a transcription factor. However, additional studies are needed to confirm the dual localization of BnMicEmUP and demonstrate its DNA binding and gene regulation capacity.

## Conclusion

*BnMicEmUP* encodes a protein with a predicted molecular mass of 19.8 kDa that is homologous to bZIP-type transcription factors. The BnMicEmUP proteins appear to be transcription factors that are localized in plastids and are involved in plant responses to biotic and abiotic environmental stresses. Seeds harvested from transgenic *Arabidopsis* plants that over-express *BnMicEmUP3* had lower germination rates than seed from untransformed control plants, and from transgenics or mutants with reduced levels of *BnMicEmUP2,3* gene expression.

## Data Availability Statement

The datasets presented in this study can be found in online repositories. The names of the repository/repositories and accession number(s) can be found in the article/Supplementary Material.

## Author Contributions

FS conducted the experiment, analyzed the data, and wrote the manuscript. KP developed the overall strategy, designed experiments, coordinated the project, and revised the manuscript. All authors contributed to the article and approved the submitted version.

### Conflict of Interest

The authors declare that the research was conducted in the absence of any commercial or financial relationships that could be construed as a potential conflict of interest.

## Footnotes

^1^https://www.arabidopsis.org/portals/masc/projects.jsp and http://www.arabidopsis.org

^2^http://www.qiagenbioinformatics.com/

^3^http://www.ncbi.nlm.nih.gov/BLAST/

^4^http://workbench.sdsc.edu/

^5^http://www.ncbi.nlm.nih.gov/BLAST/

^6^https://www.expasy.org/resources/blast

^7^http://www.ncbi.nlm.nih.gov/Structure/cdd/cdd.shtml

^8^http://www.chromDB.org

^9^http://signal.salk.edu/cgi-bin/tdnaexpress

^10^http://www.expasy.org

^11^http://planttfdb.gao-lab.org

^12^https://www.expasy.org/resources/prosite

^13^www.arabidopsis.org

^14^https://genomevolution.org/CoGe

^15^http://bioinformatics.psb.ugent.be/webtools/plantcare/html/

^16^http://www.dna.affrc.go.jp/PLACE/

^17^http://brassicadb.org/brad/index.php

^18^https://genomevolution.org/CoGe
