# Supplementary material for: Identification, Gene Structure, and Expression of BnMicEmUP: A Gene Upregulated in Embryogenic Brassica napus Microspores
Source: Front Plant Sci. 2021 Jan 11;11:576008. doi: 10.3389/fpls.2020.576008 (PMC7845737; doi:10.3389/fpls.2020.576008)
Supplement: Supplementary file 1 [file Data_Sheet_1.pdf]

Identification, Gene Structure, and Expression of *BnMicEmUP*, a Gene Upregulated in Embryogenic *B. napus* Microspores.

Shahmir F., Pauls KP

Supplementary Materials

Supplementary Tables1 Nucleotide sequences of PCR primers

	**Oligo Name**	**Forward Primer Sequence**	**Reverse Primer Sequence**	**Application**
1	At.25185 atg-gta	CATGGCTGTCAA CGCTCCGATATC	GATGGACTTC AAGAAGCTGA TTGA	Whole *BnMicEmUP* genomic and cDNA isolation
2	BnMicEmUP2,3	CCGATATCGTCT CCTGCGGCTC	CGGGACTATTC TTCGAAGGGA	Real time amplification of *BnMicemUP2,3*
3	BnMicEUP1,4	CCTATATCGTCT CACCCGCGAA	CATCAGCCTTC TCCGTCGTCGC	Real time amplification of *BnMicEmUP1,4*
4	At25185 118-735	CCTCTCCAATCC CGTTCTCCCC	TCTCTCCAACC CCGTTCTCCC	Partial isolation of *BnMicEmUP* genomic and cDNA
5	At.25185 57-217	CTCTCCAACCCC GTTCTCCCCT	TCACCAGCAG CCTTCTCCGTC G	Partial isolation of *BnMicEmUP* genomic and cDNA
6	At.25185 80-422	CTTTCGCCGGTC ATACTCCG	CTTAGCCTCGG CTGCATTACC	Partial isolation of *BnMicEmUP* genomic and cDNA
7	3utr race2	CCTATATCGTCT CACCCGCGAA	Universal primer	Isolation of the 3'-end of the cDNA
8	5utr 11 R race	Universal primer	CAATATCGTCT CCTGCGGCTCA CCCGCG	Isolation of the 5'-end of the cDNA
9	GFP fusion	CTTCAAGAAGCT GACATGGGCAA GGGCGAG	GCAGAGCTCA TCTTCACTTGT AGAGTTCATCC	Create GFP fusion protein

10	AT25185-H+ BamH1 At.25185 – C+ SacI	GGATCCAAGGA GATATAACAATG GCT GTCGTCGGCGCT CCAATA	TGGATGGACTT CAAGAAGCTG ACATGGGCAA GGGCGAGAAA CTGTTC	Amplification of *AtBnMicEmUP* or *BnMicEmUP* fragment for GFP fusion protein formation in pBI121
11	AteIF1alphaH769 AteIF1alphaC889	CTTCGTCTTCCA CTTCAGGATGT	TCAACCCTGTG GGAGCAAAG	Real time amplification of AteIF1alpha
12	At.25185 C 375 At.25185 H 274	AACGTTGAGAAA TCTGGCCTGCTG	TTCTCCAAAGC AGAGGACCTT GGT	Quick change

Supplementary Table 2 *BnMicEmUP* gene specification and accession # in Genebank.

Name	Identifier gDNA	Identifier cDNA	Accession # gDNA	Accession # cDNA	Size gDNA (bp)	Size cDNA (bp)	size Exon 1 (bp)	Size Exon 2 (bp)	Size Intron (bp)
BnMicEmUP1	8893ma1g4	5m33m4124	HQ647330	HQ660218	709	588	146	442	121
BnMicEmUP2	95353ma3g1	m23m21.1	HQ647331	HQ660217	803	594	152	442	209
BnMicEmUP3	100953ma2g1	5m33m3112	HQ647332	HQ660216	863	594	152	442	269
BnMicEmUP4		5m3m9.1		HQ660215		588			
AT1G74730					1131	891	155	441	240

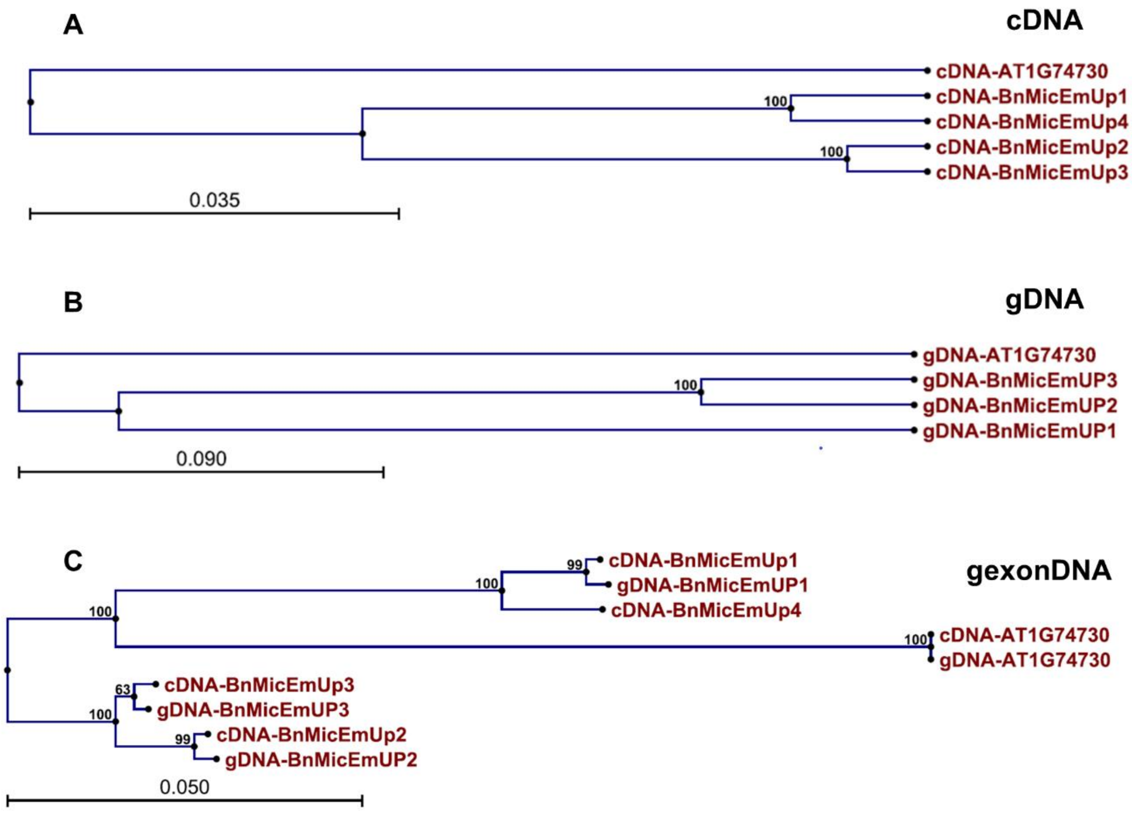

Supplementary Figure 1 Un-rooted dendrograms of *BnMicEmUPs* and *AT1G74730*.The phylogenetic trees represent the relationships between (A) gDNA, B) cDNA, (C) gexon (genomic DNA without intron) and corresponding cDNA. Phylogenetic trees were constructed by the neighbor-joining method with 1,000 bootstrap replicates using CLC software. Bootstrap values greater than 50% are shown at the branches.

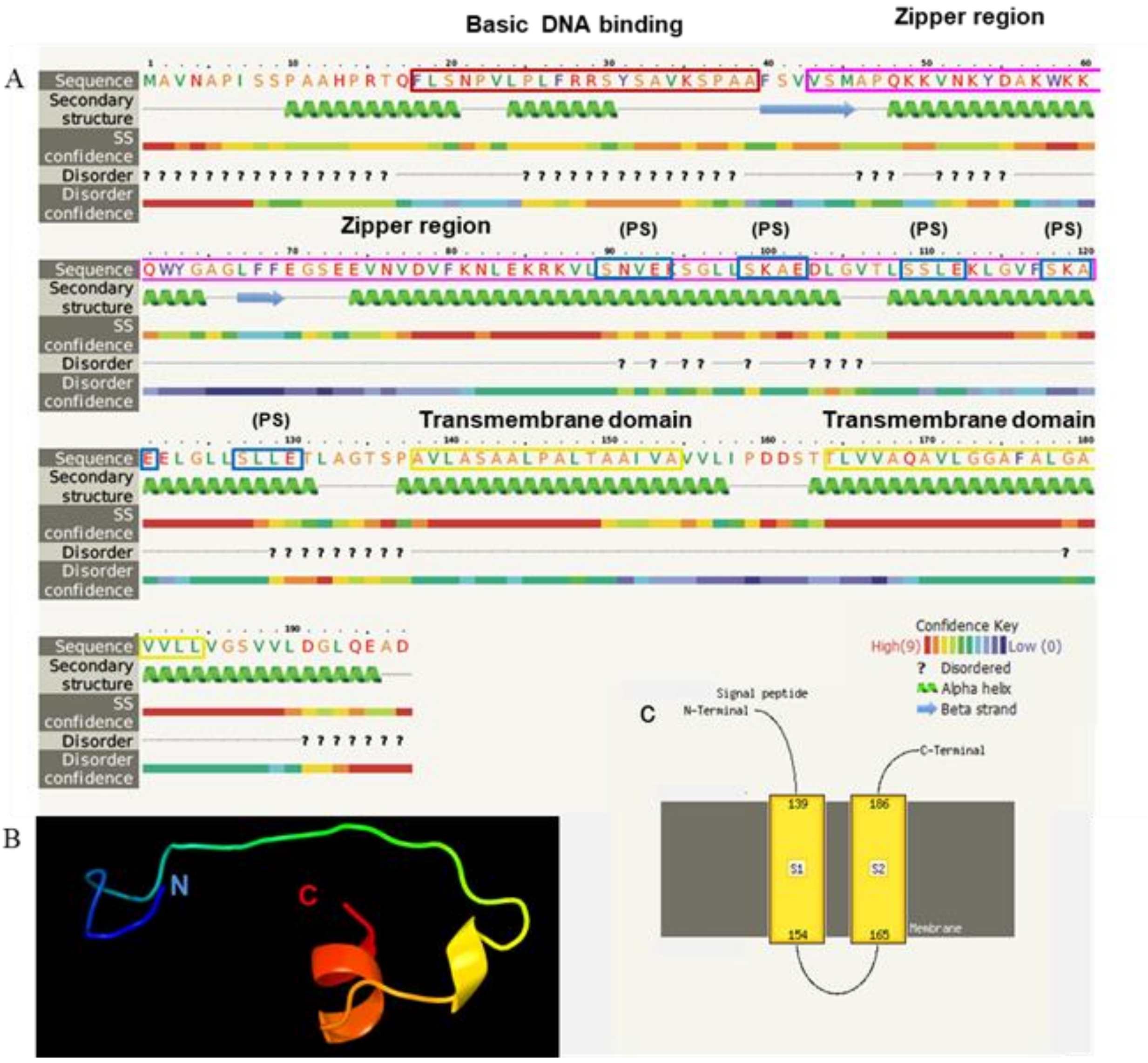

Supplementary Figure 2. The secondary structure of BnMicEmUP3 protein. **(A)** α-helix and β-strand structure. Basic DNA binding and leucine zipper (dimerization domain) are shown in black boxes. Potential casein kinase II phosphorylation sites are indicated in blue boxes(S/TxxD/E). Putative transmembrane helixes of BnMicEmUPs are shown in yellow boxes. **(B)** 3D view of BnMicEmUP3protein, images colored by rainbow N → C terminus. **(C)** Putative transmembrane helix topology of BnMicEmUP3.

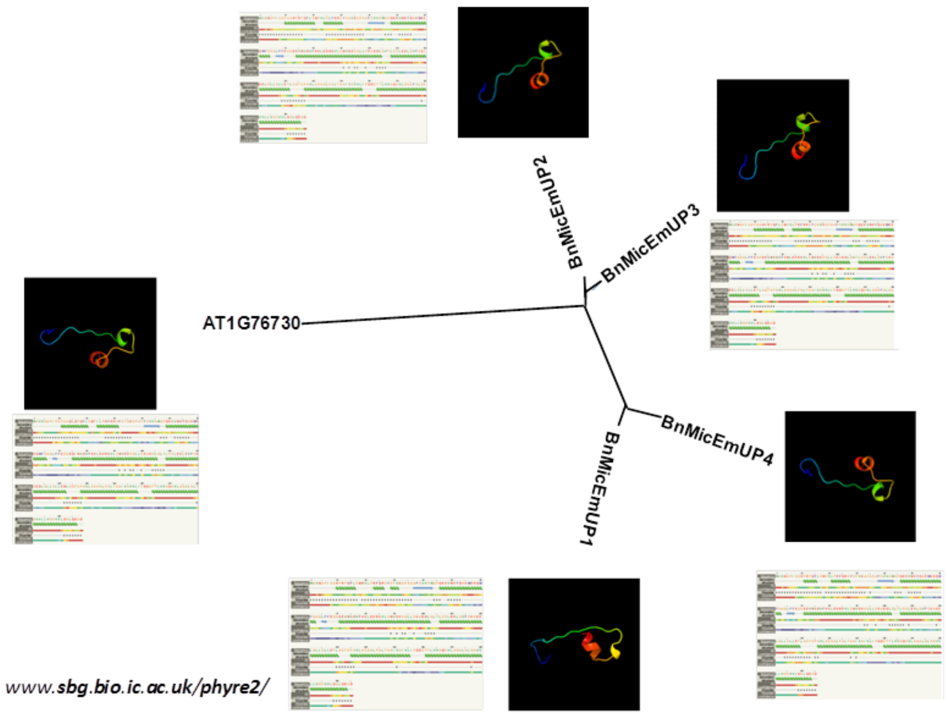

Supplementary Figure 3 Secondary structures of different BnMicEmUP proteins compared to the structure for AT1G74730. The structures where predicted by Phyr2 (Kelley et al., 2009]. The dendrogram was constructed using SDSC Biology Workbench.

Supplementary Table 3 Cis regulatory elements found in upstream sequences of *BnMicEmUP* and *AtBnMicEmUP* with their functional depiction, according to their putative function assigned from Plant CARE (http://bioinformatics.psb.ugent-be/webtools/plant care/html/, Lescot et al., 2002).

**Name of the *cis* element**	**Sequence of *cis* element**	**Function**
**Core element**		
TATA-box	TATA	Core promoter element around -30 of transcription start
CAAT-box	CAAT	Common cis-acting element in promoter and enhancer regions
**Metabolic element**		
TGA-box	TGACGTGGC	Part of an auxin-responsive element

ABRE	CACGTG	*Cis*-acting element involved in the abscisic acid responsiveness
TGACG-motif	TGACG	Cis-acting regulatory element involved in the MeJA-responsiveness
CGTCA-motif	CGTCA	Cis-acting regulatory element involved in the MeJA-responsiveness
TCA-element	GAGAAGAATA	Cis-acting element involved in salicylic acid responsiveness
GARE-motif	AAACAGA	Gibberellin-responsive element
**Binding site element**		
CCAAT-box	CAACGG	MYBHv1 binding site
**Stress element**		
TC-rich repeats	ATTCTCTAAC	*Cis*-acting element involved in defence and stress responsiveness
HSE	AAAAAATTTC	*Cis*-acting element involved in heat stress responsiveness
W box	TTGACC	*Cis*-acting element involved in pathogen responsiveness.
ARE	TGGTTT	Cis-acting regulatory element essential for the anaerobic induction
LTR	CCGAA	Cis-acting element involved in low-temperature responsiveness<br>.
**Elicitor element**		
Box-W1	TTGACC	Fungal elicitor responsive element
**Light element**		
G-box	CAGACGTGGCA	Cis-acting regulatory element involved in light responsiveness
GT1-motif	GGTTAA	Light responsive element
**Regulation element**		
O2-site	GATGACATGA	Cis-acting regulatory element involved in zein Metabolism regulation

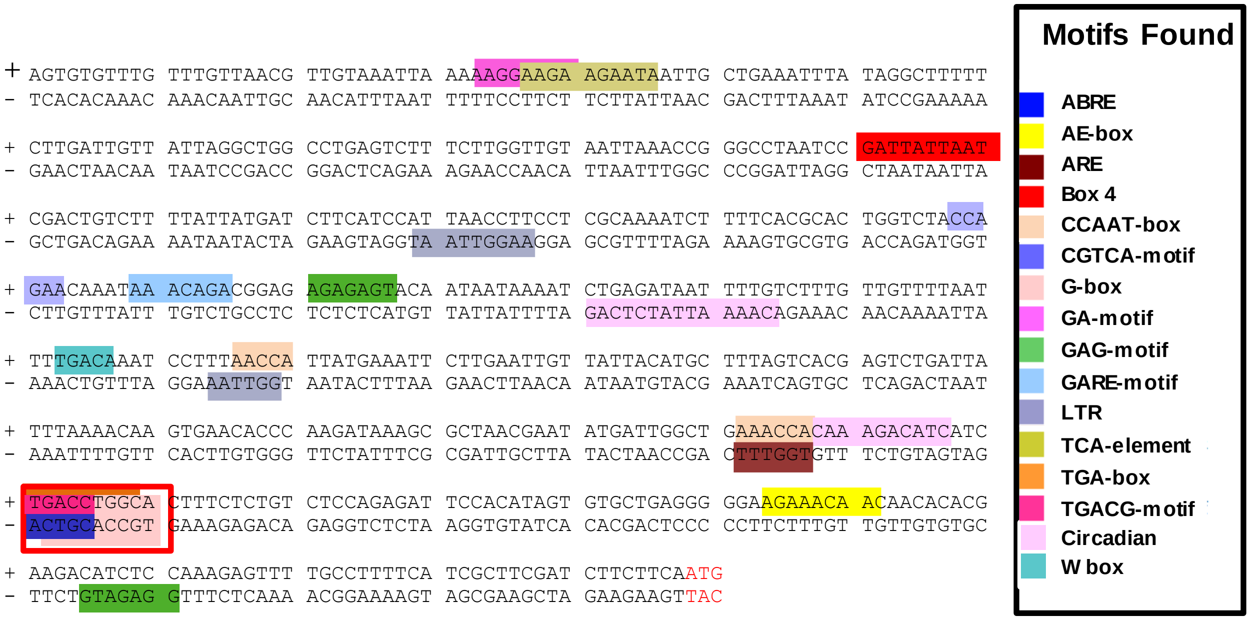

Supplementary Figure 4. Cis regulatory elements found in upstream sequence of *BnMicEmUP3* derived from Plant CARE and PLACE. (for abbreviation see Supplementary Table 3).

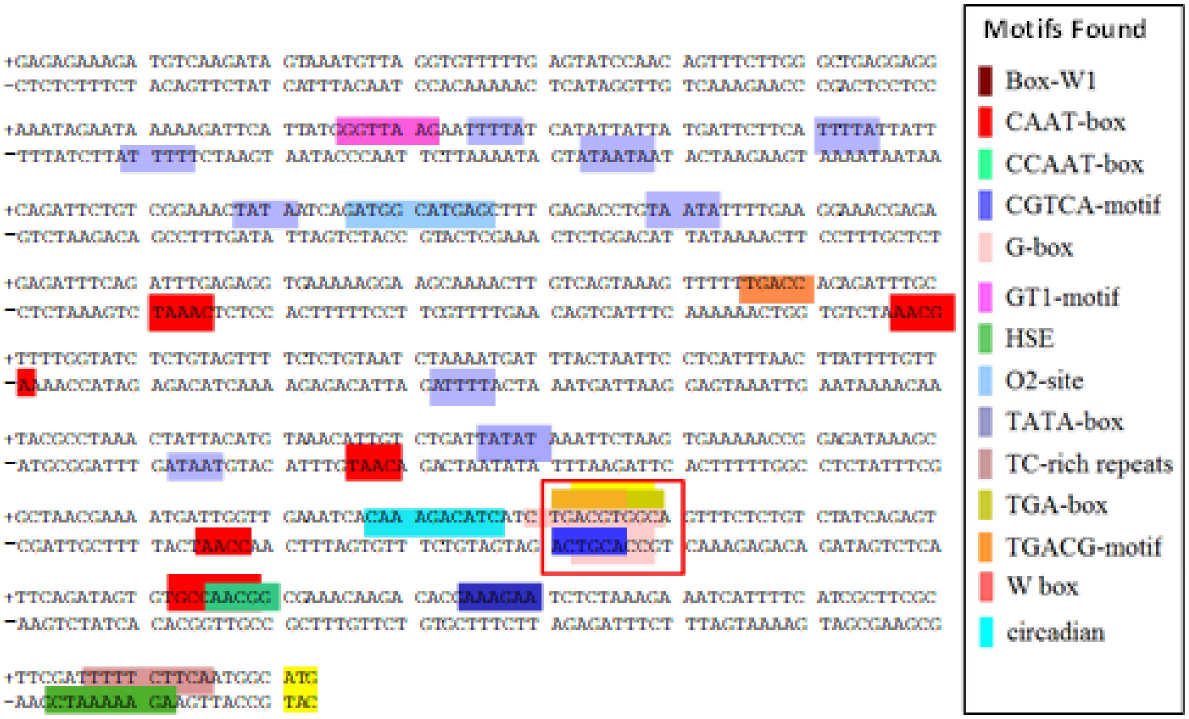

Supplementary Figure 5 Cis regulatory elements found in upstream sequence of *AtBnMicEmUP* derived from Plant CARE (http://bioinformatics.psb.ugent-be/webtools/plantcare/html/, Lescot et al., 2002).

Supplementary Table 4 Predicted localization of BnMicEmUP protein

	Program	Reference	Program description	Possible compartments	Species	Prediction
1	PSORT	Nakai and Kanehisa, 1991	Hierarchical evaluation of signal; membrane protein topology	Thylakoid membrane Mitochondrial Membrane	Plant	Chloroplast Thylakoid Membrane
2	ChloroP	Emanuelsson et al., 1999	Signal sequence	CTP 0.546	Plant	Chloroplast l

3	TargetP	Emanuelsson et al., 2000	SignalP; additional signal predictors	CTP 0.653 MTP 0.529	Plant	Chloroplast high reliability
4	PCLR	Schein et al., 2001	Signal sequence	CTP.0.97	Eukaryote	Chloroplast
5	ESL Pred	Bhasin and Raghava, 2004	Amino acid comparison Support vector machine	Mitochondria 53%	Animal	Mitochondria
6	Predotar	Small et al., 2004	Signal sequence	Plastid 0.93	Eukaryote	Plastid
7	HSL Pred	Garg et al., 2005	Amino acid comparison Support vector machine	Subcellular Localization 52%	Human	Cytoplasm Protein
8	SLP-Local	Matsuda et al., 2005	Amino acid comparison Support vector machine	Mitochondria 0.87	Plant	Mitochondria
9	CELLOv2.5	Yu et al., 2006	Support vector machine	Chloroplast 1.82	Eukaryote	Chloroplast
10	Plant-PLoc	Chou and Shen, 2007	Gene ontology (GO) Sequence similarity	Chloroplast	Plant	Chloroplast
11	BaCelLo	Pierleoni et al., 2006	Amino acid comparison Support vector machine	Chloroplast	Plant	Chloroplast

A

B

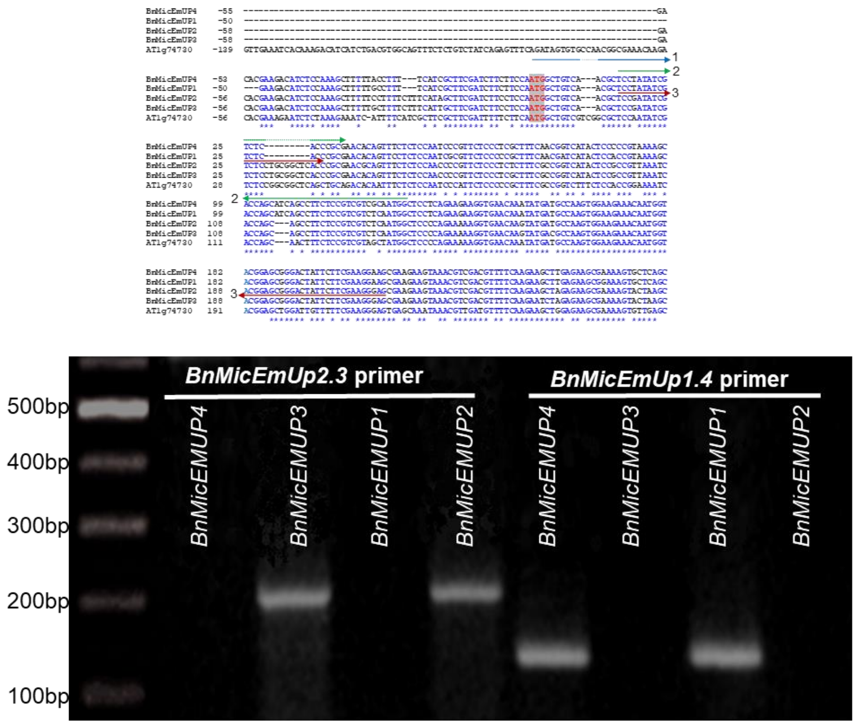

Supplementary Figure 6. PCR products generated from four different forms of *BnMicEmUP* topo clones using *BnMicEmUP2,3* (left) and *BnMicEmUP1,4* primers (right*BnMicEmUP1,4* primer amplified genes of *BnMicEmUP1* and *BnMicEmUP4*; *BnMicEmUP2,3* primer amplified genes of *BnMicEmUP2* and *BnMicEmUP3*). These primers were used for real time PCR gene expression studies.

a

b

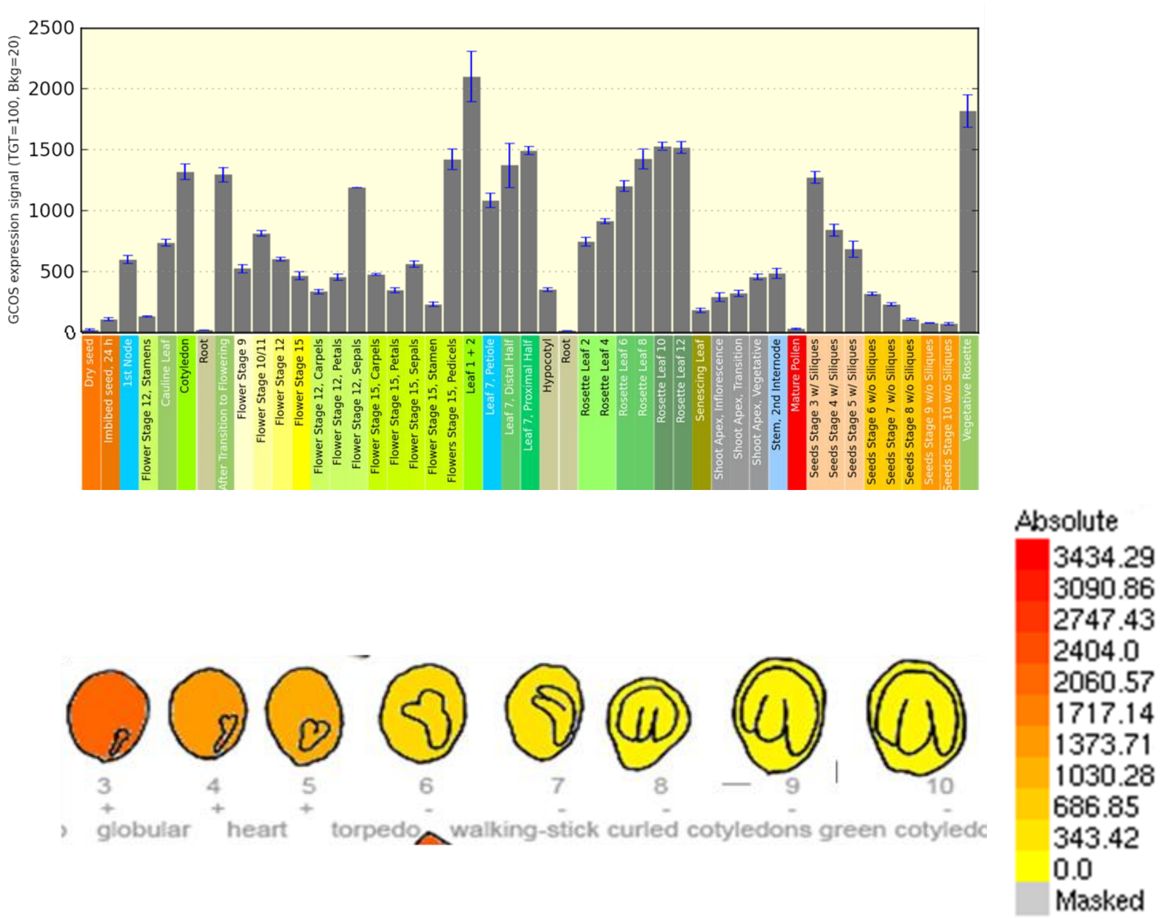

Supplementary Figure 7 Expression profile of *AtBnMicEmUP* (AT1G74730) as predicted by the *Arabidopsis* eFP browser (Winter et al., 2007). (A) Chart of expression levels in different tissues. (B) Expression pattern of *BnMicEmUP During* embryo development.
